# Impact of Bi Doping into Boron Nitride Nanosheets on Electronic and Optical Properties Using Theoretical Calculations and Experiments

**DOI:** 10.1186/s11671-021-03542-x

**Published:** 2021-05-12

**Authors:** Muhammad Ikram, Muhammad Wakeel, Jahanzeb Hassan, Ali Haider, Sadia Naz, Anwar Ul-Hamid, Junaid Haider, Salamat Ali, Souraya Goumri-Said, Mohammed Benali Kanoun

**Affiliations:** 1 Solar Cell Applications Research Lab, Department of Physics, Government College University Lahore, Lahore, 54000 Punjab Pakistan; 2 Department of Physics, Riphah Institute of Computing and Applied Sciences (RICAS), Riphah International University, 14 Ali Road, Lahore, Pakistan; 3 Department of Clinical Medicine and Surgery, University of Veterinary and Animal Sciences Lahore, Lahore, 54000 Punjab Pakistan; 4 Tianjin Institute of Industrial Biotechnology, Chinese Academy of Sciences, Tianjin, 300308 China; 5 Core Research Facilities, King Fahd University of Petroleum and Minerals, Dhahran, 31261 Saudi Arabia; 6 College of Science, Physics Department, Alfaisal University, P.O. Box 50927, Riyadh, 11533 Saudi Arabia; 7 Department of Physics, College of Science, King Faisal University, P.O. Box 400, Al-Ahsa, 31982 Saudi Arabia

**Keywords:** Boron nitride, Bi-doped boron nitride, Nanosheets, Antimicrobial, HR-TEM

## Abstract

In the present work, boron nitride (BN) nanosheets were prepared through bulk BN liquid phase exfoliation while various wt. ratios (2.5, 5, 7.5 and 10) of bismuth (Bi) were incorporated as dopant using hydrothermal technique. Our findings exhibit that the optical investigation showed absorption spectra in near UV region. Density functional theory calculations indicate that Bi doping has led to various modifications in the electronic structures of BN nanosheet by inducing new localized gap states around the Fermi level. It was found that bandgap energy decrease with the increase of Bi dopant concentrations. Therefore, in analysis of the calculated absorption spectra, a redshift has been observed in the absorption edges, which is consistent with the experimental observation. Additionally, host and Bi-doped BN nanosheets were assessed for their catalytic and antibacterial potential. Catalytic activity of doped free and doped BN nanosheets was evaluated by assessing their performance in dye reduction/degradation process. Bactericidal activity of Bi-doped BN nanosheets resulted in enhanced efficiency measured at 0–33.8% and 43.4–60% against *S. aureus* and 0–38.8% and 50.5–85.8% against *E. coli*, respectively. Furthermore, In silico molecular docking predictions were in good agreement with in-vitro bactericidal activity. Bi-doped BN nanosheets showed good binding score against DHFR of *E. coli* (− 11.971 kcal/mol) and *S. aureus* (− 8.526 kcal/mol) while binding score for DNA gyrase from *E. coli* (− 6.782 kcal/mol) and *S. aureus* (− 7.819 kcal/mol) suggested these selected enzymes as possible target.

**Supplementary Information:**

The online version contains supplementary material available at 10.1186/s11671-021-03542-x.

## Introduction

Various chemicals, organic compounds, and industrial waste give rise to environmental pollution those results in serious consequences for human, animal, and aquatic life [1, 2]. Due to this reason, innovative and environmentally-friendly wastewater treatment technologies are in growing demand [3, 4]. Millions of people lose their lives each year due to contaminated water [5, 6]. Almost annually dyes utilization is ~ 10,000 in industrial sectors; among them, a prominent source is methylene blue (MB) used 10–15% in the atmosphere and aquatic life [7–10]. MB is a simple aniline dye with the molecular formula C_16_H_18_N_3_SCl that is widely used for dying cotton, wool, and silk as well as treating methaemoglobinemia and cyanide poisoning. It is used by biologists to stain tissue samples and to detect nucleic acids. Regardless, this dye has a number of negative effects on both humans and wildlife. As a result, removing dyes from drainage is important for the welfare of humans and aquatic life [11, 12].

Conventional methods used for the elimination of various contaminants from water include precipitation, electrolysis, flocculation, photocatalysis, membrane filtration, ions exchange, adsorption, reverse osmosis and biological treatment [13, 14]. In these methods, catalytic activity (CA) is widely used due to its cost-effective and environmentally sustainable approach [15]. CA comprises of reducing agent and nanocatalyst which is the prepared sample to degrade synthetic dye such as MB which is the part of present study [16–18].

Increasing requirements to purify wastewater has led to the development and use of a new class of nanomaterials known as two-dimensional materials (2D-Mats). The utilization of these materials was spurred by the discovery of graphene [19–21]. At present, a variety of 2D-Mats has been synthesized including molybdenum disulfide (MoS_2_) and MXene (Dirac 2D-Mats) [22, 23]. Boron nitride is considered a promising class of MXene class [24, 25]. BN nanosheets possess several interesting properties including dielectric performance, chemical and thermal stability, deep ultraviolet and direct bandgap energy making it suitable for use in a variety of applications especially for water treatment and antimicrobial activities as well [24, 26, 27]. To state these tasks bismuth-rich strategies or doping with different transition metal elements (i.e. Bi) are the most accessible methodologies. Bi has an extraordinary appearance compared to others showing color-gray-white with a reddish tinge (pinkish stain). Bi forms chemical compounds in oxidation states of + 3 and + 5. Bi compounds are used as nanocatalyst for wastewater treatment and it is also a good antimicrobial agent when it is utilized as dopant in 2D-Mats such as BN as discussed above [28–31].

In addition to above, BN nanosheets can also be utilized in biomedical sector as a antimicrobial agent with an aim to protect against various bacteria’s [32]. Mastitis is distinguished by physicochemical and patho-biological alterations in udder parenchyma tissues, containing direct economic effects worldwide. Humans are at high risk of suffering zoonotic diseases such as leptospirosis, streptococcal sore throat, brucellosis, and tuberculosis due to mastitic milk consumption [33]. Generally, infectious etiological agents that involve bacteria and viruses are categorized into two classes. The first category includes *Staphylococcus aureas* (*S. aureus*), *Coliform*, *Corynebacterium*, *Streptococci* and *Escherichia coli (E. coli)*. The second category comprises *Corynebacterium bovis* and *coagulase-negative Staphylococci* [2, 32, 34, 35]. Among these, more prominent is *Methicillin-Resistant Staphylococcus aureus* (MRSA) as it contributes to a large number of deaths worldwide. Resistance to antibiotics has emerged in Gram-positive and Gram-negative pathogenic bacteria, posing a serious risk to human health [36]. Besides, diarrheal illness caused by the presence of *E. coli* bacteria in water results in 1.3 million deaths of children below the age of five annually. Being an antibacterial agent, BN protects from these harmful pathogens [37]. Due to Bi's biocompatibility, the synthesis and usage of Bi in various forms such as Bi salts, NPs, and nanomaterials as antimicrobials has received a huge of attention [38]. Infections caused by Helicobacter pylori (H. pylori) are currently treated with a mixture of Bi organic salts and antibiotics [39, 40]. The oxidative stress created by nanostructure depends upon its size; concentration and shape as Small-sized nanostructure produce reactive oxygen species (ROS) that bind more efficiently within bacterial membrane within implants resulting in extrusion of cytoplasmic contents and damaging bacterial DNA, proteins, and enzymes [41–43]. Besides ROS production, the strong cationic interface of nanostructures with negatively charged bacteria cell membrane parts results in superior bactericidal activity at high concentrations encouraging bacteria cell collapse [44, 45].

In the present study, BN nanosheets were prepared with chemical exfoliation technique while bismuth (Bi) was incorporated as a dopant using hydrothermal technique. CA of the synthesized material was determined in terms of reduction of harmful MB. Furthermore, antibacterial activity was assessed against *E. coli* and *S. aureus.* To identify possible mechanism of action, molecular docking studies of Bi-doped BN nanosheets were performed against dihydrofolate reductase (DHFR) enzyme from Folate biosynthetic pathway alongside DNA gyrase from nucleic acid biosynthetic pathway belonging to both *E. coli* and *S. aureus*. The first-principles density functional theory calculations were performed to computed the stability structure, electronic and optical properties of pristine and Bi-doped BN nanosheet.

## Methods

The current study was impact of Bi-doped BN nanosheets on electronic and optical properties using theoretical calculations and experiments: dye degradation, anti-bactericidal behavior and molecular docking analysis.

### Experimental Details

BN bulk powder (98%), dimethylformamide (DMF) were procured from Sigma-Aldrich, Germany. Bismuth nitride pentahydrate Bi(NO_3_)_3_·5H_2_O (98%) from BDH laboratory supplies Poole, UK. All received chemicals were utilized without purification treatment.

**Fig. 1:**
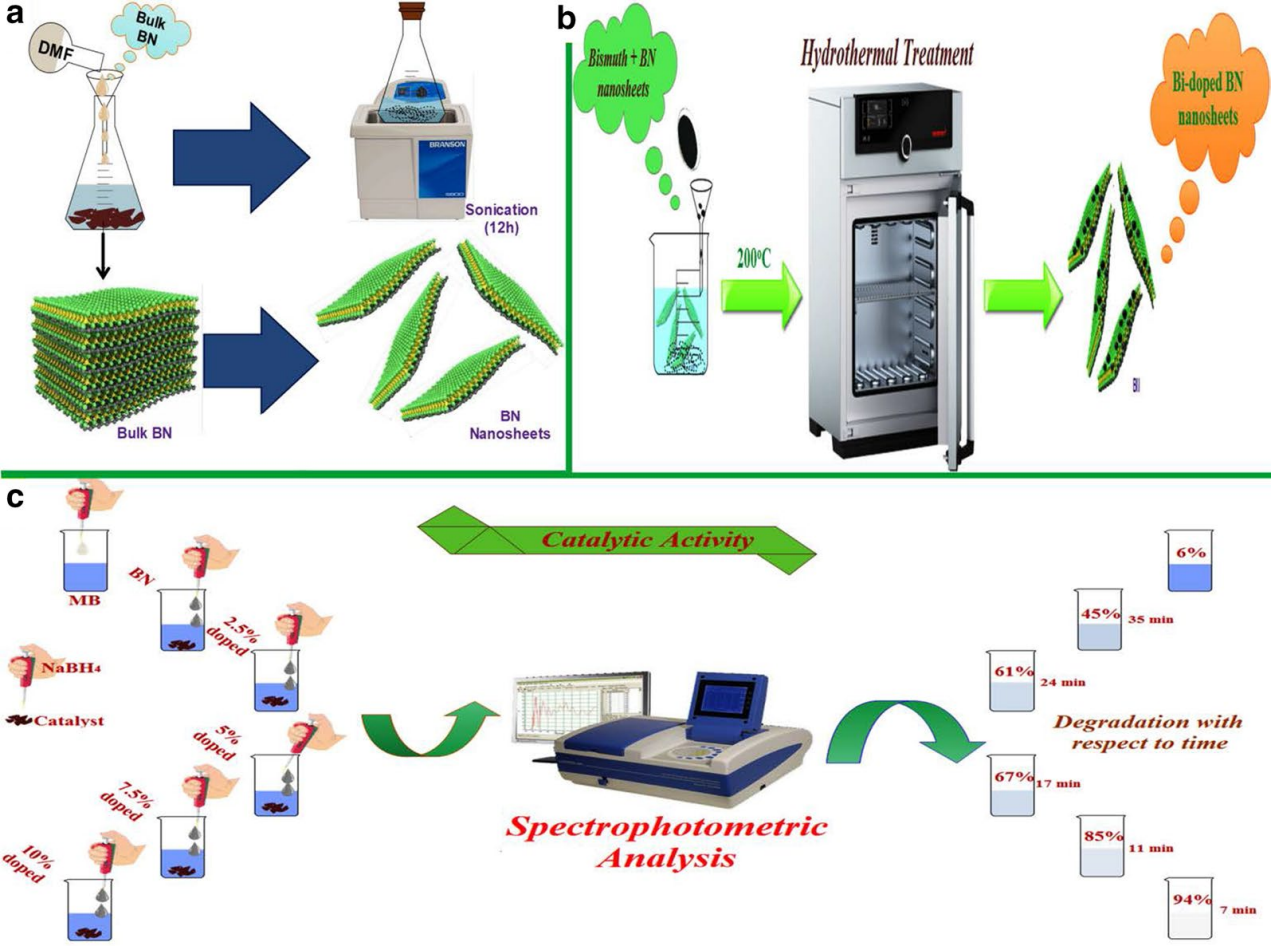
Schematic representation of **a** liquid phase exfoliation of bulk BN; **b** hydrothermal synthesis; **c** catalytic activity

To produce BN nanosheets, liquid phase exfoliation of bulk BN was carried out. 200 mg of bulk BN powder was dissolved in DMF (50 ml) and stirred for 15 min. Subsequently, the dissolved solution was ultrasonicated for 12 h at 50 °C as illustrated in Fig. 1a. This ultrasonicated suspension was centrifuged at 3500 rpm for 20 min [46]. Collected nanosheets were doped with Bi using bismuth nitride pentahydrate Bi(NO_3_)_3_·5H_2_O as a source of Bi using hydrothermal method. Various Bi dopant concentrations (2.5, 5, 7.5 and 10 wt%) were added in BN nanosheets separately at fixed ratios (0.025:1, 0.05:1, 0.075:1 and 0.1:1) in a Teflon vessel and transferred to autoclave for 12 h at 200 °C as shown in Fig. 1b. After that, autoclave was cool down and obtained product was washed repeatedly using cleaning agents such as ethanol and deionized water to eliminate impurities, and solution was dried at 100 °C in a vacuum oven.

Van der Waals attractions are the predominant forces among the stacked layers of bulk boron nitride. These Van der Waals interactions must be overcome to exfoliate stacked layers. This was performed using intercalation of organic solvent into layers followed by introduction of mechanical forces attained from ultra-bath sonicator. Solvents whose surface tension matches with that of boron nitride are the ideal solvents for well dispersion of bulk boron nitride, as they minimize the interfacial tension between the solvent and boron nitride. That is why we have adopted DMF as its surface tension is matches with graphene (37.1 m J m^−2^) and BN is analogous to graphene so it is a quite suitable solvent to disperse boron nitride nanosheets [47].

Ultrasonic radiations travel through the medium, solvent molecules compress and stretch i.e. starts oscillating about their mean positions resulting in development of high pressure regions that can be named as compression and negative pressure regions as stretching. When negative pressure is not large enough to hold liquid molecules intact, then breakdown of liquid takes place forming voids (cavitation bubbles). These cavitation bubbles will collapse violently in high pressure regions and behave like micro reactors, produce local temperature of several thousand degrees and high pressure of several hundred atmospheres which is sufficient to overcome the inter-sheet attractive forces and hence induces exfoliation [48, 49].

### Catalytic Activity

Catalytic potential was evaluated by undertaking MB dye degradation in the presence of sodium borohydride (NaBH_4_) which serves as reducing agent. Firstly, an appropriate amount of dye and reductant was diluted in deionized water to prepare an aqueous solution. Catalytic experiment was carried out by utilizing all prepared samples as nanocatalyst. Dye degradation was measured by adding NaBH_4_ solution (600 μl) to MB (10 ml) in a quartz cell. It is worth mentioning here that NaBH_4_ is unable to degrade the dye, therefore it serves as a reducing agent only. Furthermore, each catalyst (4 mg) was added separately into a precursor solution to investigate catalytic efficiency for dye degradation. Reduction of dye was measured by taking absorption spectra in the range 450–750 nm with a UV–vis spectrophotometer. In this regard, decolorization of methylene blue is considered an indication of successful dye degradation. Schematic illustration of performed activity with pure BN and various doping concentrations is shown in Fig. 1c. The illustration to the left-hand side signifies performed activity while that to the right shows degradation concentration concerning the time after taking absorption spectra with UV–Vis spectroscopy.

### Antimicrobial Activity

In-vitro assessment of antimicrobial potential of Bi-doped BNNS was accomplished through well diffusion method by swabbing 1.5 × 10^8^ CFU/mL of G+ve and –ve bacterial isolates on MSA and MA, respectively as shown in Additional file 1: Fig. S1. Various ratios of Bi-doped BNNS (500 μg/50 μl) and (1000 μg/50 μl) were inoculated as low and high dose in wells (6 mm) on MSA and MA plates Additional file 1: Fig. S1. Ciprofloxacin (5 μg/50 μl) and DIW (50 μl) were labeled as positive (+ve) and negative (−ve) controls. Anti-bacterial evaluation was proven by inhibition zones (mm) measurements using Vernier caliper after overnight incubation of Petri dishes at 37 °C [50]

### Materials Characterization

X-ray diffractometer (XRD) from Bruker (D_2_ Phaser, USA) equipped with Cu-K$\alpha$ (*λ* = 0.154 nm) was used with diffraction angle (2θ) range from 10° to 60° with 0.05/min scan rate to determine the structural characteristics of synthesized material. Fourier transform infrared (FTIR) spectroscopy (Perkin Elmer spectrometer) with wavenumber accuracy within ± 0.01 cm^−1^ was employed to outline IR fingerprints. Optical properties were evaluated using GENESYS-10S UV–Vis with a scan rate of 5 nm/s and absorption spectra range from 200–800 nm and photoluminescence study was undertaken with JASCO FP-8200 spectrofluorometer with scan rate 10 nm/s. Surface morphology and microstructure were studied using field emission scanning electron microscope (FESEM model JSM 6460LV) coupled with energy dispersive x-ray (EDS) spectrometer and JEOL JEM 2100F high-resolution transmission electron microscope (HR-TEM).

### Computational Details

Our first-principles calculation was conducted by comprehensive framework of the DFT as implemented in the QuantumATK software [51] using local combination of the atomic orbitals (LCAO) approach. The exchange–correlation functional was conducted by Perdew, Burke, Ernzerhof (PBE) connecting with the generalized gradient approximation (GGA) [52]. The norm-conserving PseudoDojo [53] pseudopotential was employed for describing the interaction between electrons and ions, and the valence electrons The Brillouin region was performed employing Monkhorst–Pack's special k-point grid of 4 × 4 × 1 for structural relaxation and 7 × 7 × 1 for electronic property calculations. The calculation of self-consistent field (SCF) was taken into account a tolerance limit of 10^−6^ Ha for energy convergence. The geometry structure and ion relaxations were carried out by using the limited-memory Broyden–Fletcher–Goldfarb–Shanno (LBFGS) algorithm, including the force on each atom less than 0.05 eV/Å. On account of the strong relativistic effect owing to presence of heavy Bi dopant, spin-orbital coupling (SOC) contribution has been considered in the calculation of the electronic structures.

## Results and Discussion

### Structure and Electronic Properties

**Fig. 2:**
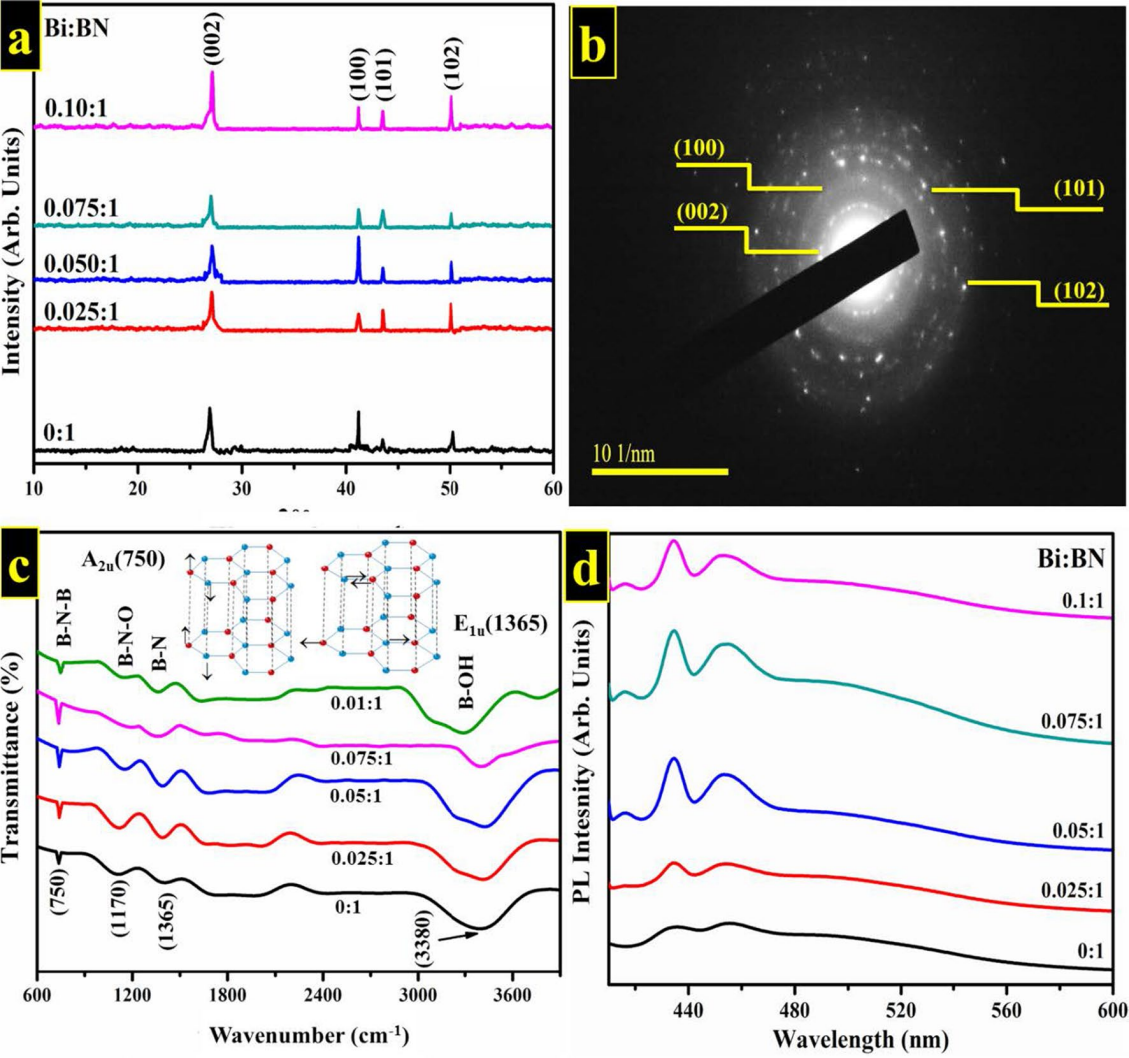
**a** XRD patterns of host and Bi-doped BN nanosheets with various concentrations (2.5, 5, 7.5 and 10 wt%); **b** SAED pattern obtained from BN nanosheets; **c** FTIR spectra; **d** PL spectra

XRD was employed to investigate phase identification, crystallinity, and crystallographic planes of control and Bi-doped BN nanosheets as illustrated in Fig. 2a. The XRD reflections identified at 2θ values of ~ 26.9°, 41.3°, 43.46°, and 50.2° were respectively indexed as (002), (100), (101), and (102) crystallographic planes. Detected crystallographic planes harmonized well to standard spectrum (JCPDS reference #00-034-0421) [54, 55]. Sharpness and peak intensity suggest formation of BN thin layers and weak stacking of NS in preferred c-direction [46]. Peak shift concerning diffraction angle was detected in XRD reflections, which suggests incorporation of dopant. Interlayer spacing (*d*-value) of characteristic (*d*_002_) reflection was found ~ 0.34 nm as evaluated through Bragg’s law $(n\lambda =2d\mathrm{sin}\theta )$ and correlates well with HR-TEM results (see Additional file 1: Fig. S4). This d-value of corresponding plane is characterized by significant features pertaining to adsorption properties and molecular transport of BN that serves to enhance its catalytic performance [56]. Corresponding SAED profiles of bare BN represented in Fig. 2b consist of bright circular rings that indicate high crystallinity of sample. These detected rings agree well with XRD patterns and standard data [26, 57, 58]. The surface morphology of the synthesized material was explored using FESEM and further confirmed through HR-TEM analysis. Interlayer spacing was evaluated with Gatan digital micrograph software using HR-TEM images, which was found to be consistent with XRD results. The purity of prepared product was ascertained by analyzing elemental composition through EDS spectroscopy as illustrated in Additional file 1: Figs. S5 and S6.

FTIR was employed to investigate IR fingerprints of host and Bi-doped BN nanosheets as shown in Fig. 2c. Obtained spectra display two characteristic peaks originating from BN at 750 and 1365 cm^−1^ can be attributed to B–N–B (bending vibrations) as well as B–N (stretching vibration). These core peaks are related to A_2u_ mode (out-of-plane) as well as E_1u_ mode (in-plane) [56, 59]. As mentioned A_2u_ mode is an out-of plane mode that corresponds to the energy of 96.4 meV, whilst the E_1u_ corresponds to in-plane modes that further splits into two modes first one is longitudinal optical mode of E_1u_^TO^ and secondly transverse optical E_1u_^LO^ with energies of 169.4 and 199.6 meV respectively, due to the long-range Coulomb interactions as pointed out by Michel and Verberck [60]. In their work, they compared two phonon dispersion relations that were calculated without and with the long range Coulomb force, respectively. The change among the two calculations corresponds to LO-TO splitting. Owing to Coulomb interaction which breaks the symmetry field in BN leading to the divide the longitudinal and transverse optical phonons [61]. A schematic illustration of these modes is shown in Fig. 2c. An additional peak was detected at 1170 cm^−1^ is associated with the stretching vibration of boron oxynitride (N–B–O). Broad band at 3433 cm^−1^ corresponds to O–H stretching vibration [62].

PL spectroscopy was used to confirm the excitons migration, transfer, and recombination in samples as shown in Fig. 2d. Extracted spectra were marked with excitation wavelength i.e. *λ*_ex_ = 390 nm and corresponding emission wavelength *λ*_em_ = 420 nm. Since nanoscale materials are relatively sensitive to excitation wavelength, emission spectra are based on the value of *λ*_ex_ [59]. PL spectra of undoped and Bi-doped BN nanosheets displayed asymmetric peaks located at ~ 420 nm onwards. These detected asymmetric peaks in PL spectra suggest the existence of luminescent species and/or multi-fluorophores. Literature studies suggest that the presence of species such as boron–oxygen is regarded as novel luminescence centers in BN system [63]. Luminescence founds around 460 nm represent electronic transition commencement. This electronic transition involves individual/mutual transition lies between 2p states of BN bands [64]. Excitation of an electron (e^−^) from valance to conduction band serves to enhance intensity of luminescence and energy of excitation light. This transition at 460 nm corresponds to energy peak at ~ 2.7 eV [65]. It is worth mentioning that samples were prepared via same quantity, growth rates, as well as durations etc., but the somewhat difference in intensities of all the samples for PL spectra may be attributed to less h-BN domains per unit area that are taking part in luminescence [66]. Maximum recombination and separations of excitons correspond to highest and lowest intense peaks in PL spectra respectively [67].

**Fig. 3:**
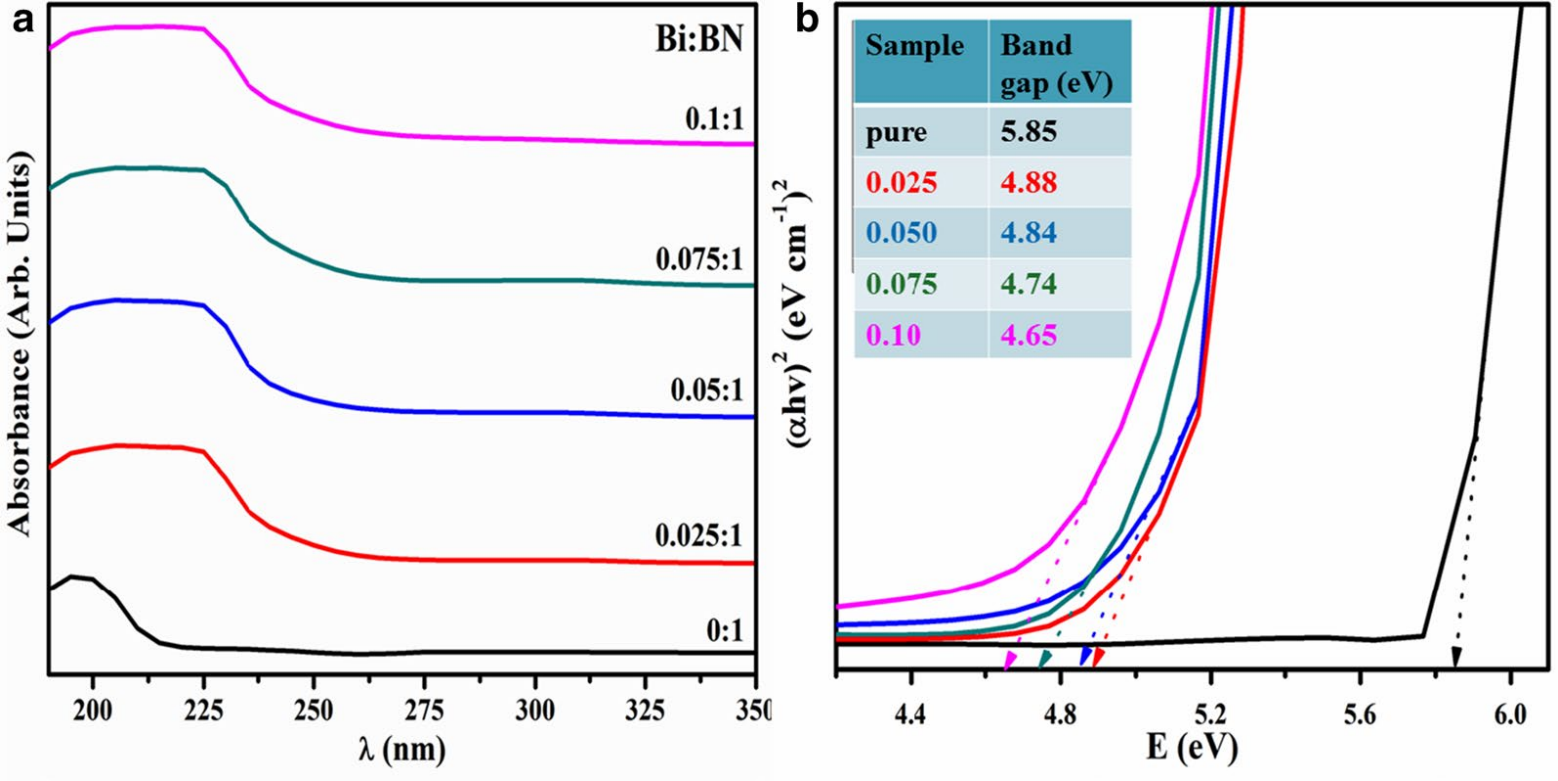
**a** UV–Vis spectra of host and Bi-doped BN nanosheets; **b** bandgap energy analysis using Tauc plot

Optical properties of host BN and Bi-doped BN nanosheets were ascertained through absorption spectra obtained using UV–Vis spectroscopy. Appearance of absorption in near UV region was observed as illustrated in Fig. 3a. The maximum absorption for pure BN nanosheets was detected around 200 nm which is known as near UV region that corresponds to the bandgap energy of ~ 5.85 eV. With the incorporation of Bi the maximum absorption edge is moved towards higher wavelength that indicates the redshift in optical spectra that causes to reduce the bandgap energy. Bandgap energy was estimated using Tauc equation which is represented in Eq. 1. Tauc plot in as displayed in Fig. 3b represents bandgap energy is reduced upto 4.65 eV. Besides these, no additional absorption towards lower or higher energy level was detected for pure, 2.5, and 5% Bi-doped samples, which suggests the existence of dense structural defects. Whereas for 7.5 and 10% Bi-doped samples very minor absorption around 330 is observed that is also verified and explained in the simulated optical absorption spectra analysis (see Fig. 6) [62, 68, 69]. According to the literature, bulk BN exhibits bandgap energy of 5.2–5.4 eV while bi/multilayer nanosheets possess bandgap energy of 5.56–5.92 eV [26]. These observations suggest that as-prepared BN nanosheets possess bi/multilayers configuration. Bandgap energy of all samples displayed in Fig. 3b and estimated through Tauc Eq. (1) is expressed as follows:

1 $$\alpha h\nu = {K\left(h\nu - {E}_{g}\right)}^{n}$$

In the above equation, *α* indicates absorption coefficient which is equal to $\alpha =\mathrm{log}(T/d)$ where *T* is transmission and *d* is path length. Further, value of exponent (*n*) is associated with electronic nature of *E*_g_ and corresponds to direct allowed transitions (1/2), indirect allowed transitions (2), direct forbidden transitions (3/2) and indirect forbidden transitions (3), respectively. However, transition data allows to best linear fit in band edge area if *n* = 1/2. *E*_g_ is characteristically measured by assessing of (*αhν*)^1/*n*^ vs hν plots. Linear trend acquired from Eq. 1 is modeled as the tangent of plot near point of maximum slope region. Here, hν equals photon energy (*E*), *K* is absorption index and *E*_g_ is band gap energy (eV) [26].

These experimental findings are supported by first principle-based DFT calculations. The model of undoped and Bi-doped BN monolayers is built using the supercell method with periodic boundary conditions. A 7 × 7 supercell was used for pristine and Bi-doped BN monolayer to assure minimal interaction of Bi with its neighboring images. A 15 Å vacuum layer was used along the direction perpendicular to the plane of the monolayer. The dopant concentrations of 2.04%, 4.08%, and 6.1% have been modeled by substituting one, two, and three Bi atoms in the BN monolayer supercell at B sites, as shown in Additional file 1: Fig. S6. To examine the stability of Bi dopant with different concentration, we estimated the binding energies using the following equations [70, 71]:

2 $${E}_{\mathrm{b}}={E}_{\mathrm{supercell}}-{E}_{\mathrm{V}}-{E}_{\mathrm{TM}}$$

whereby, *E*_supercell_, *E*_V*,*_ and *E*_TM_ refer to the total energy of the doped BN, host material with cation vacancy, and the isolated metal atom. It is found that the value of *E*_b_ for different dopant concentrations changes strongly from −4.0 to − 7.71 eV.

**Fig. 4:**
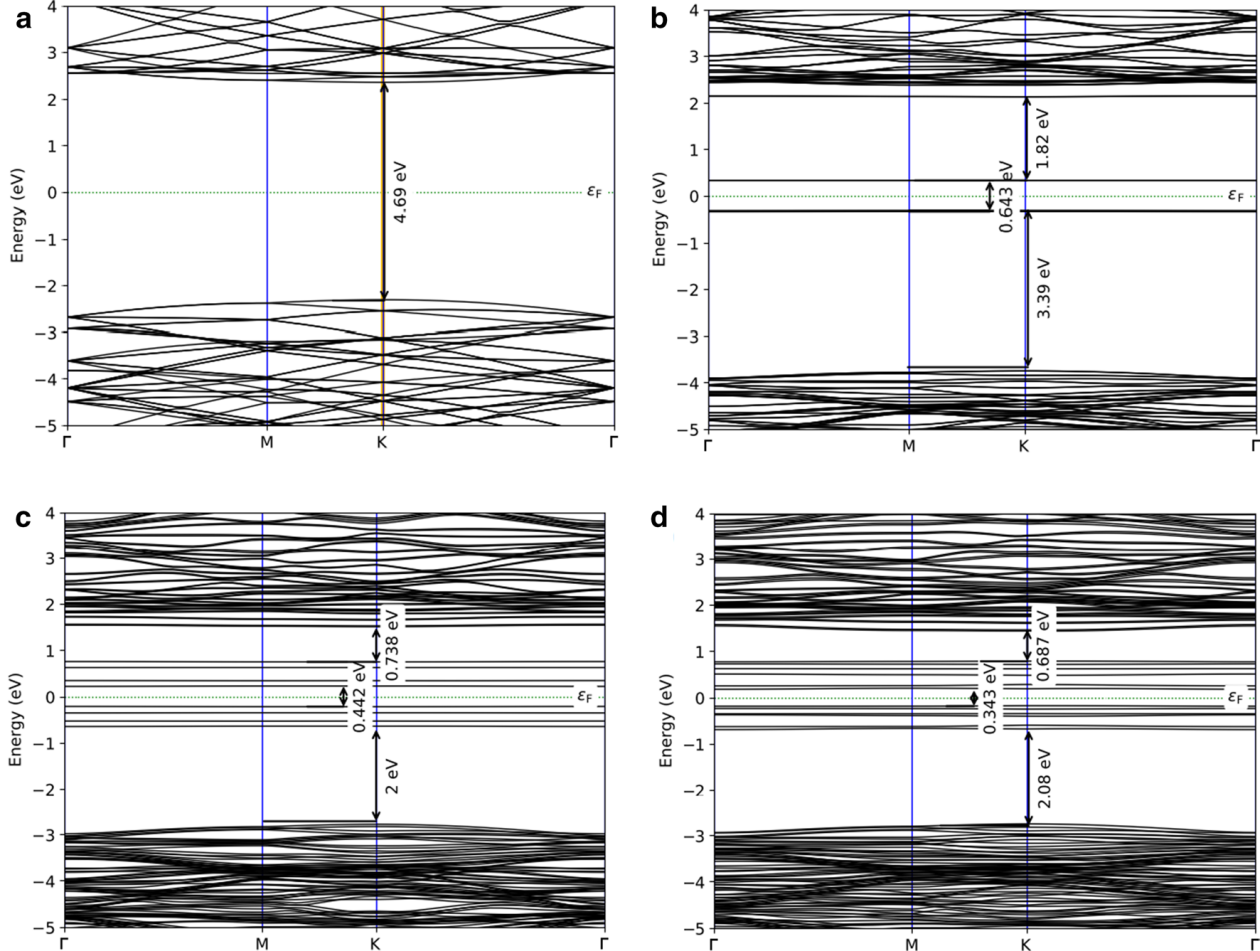
The computed electronic band structure of **a** pristine BN and Bi-doped monolayers with concentrations **b** 2.04%, **c** 4.08%, and **d** 6.1%

**Fig. 5:**
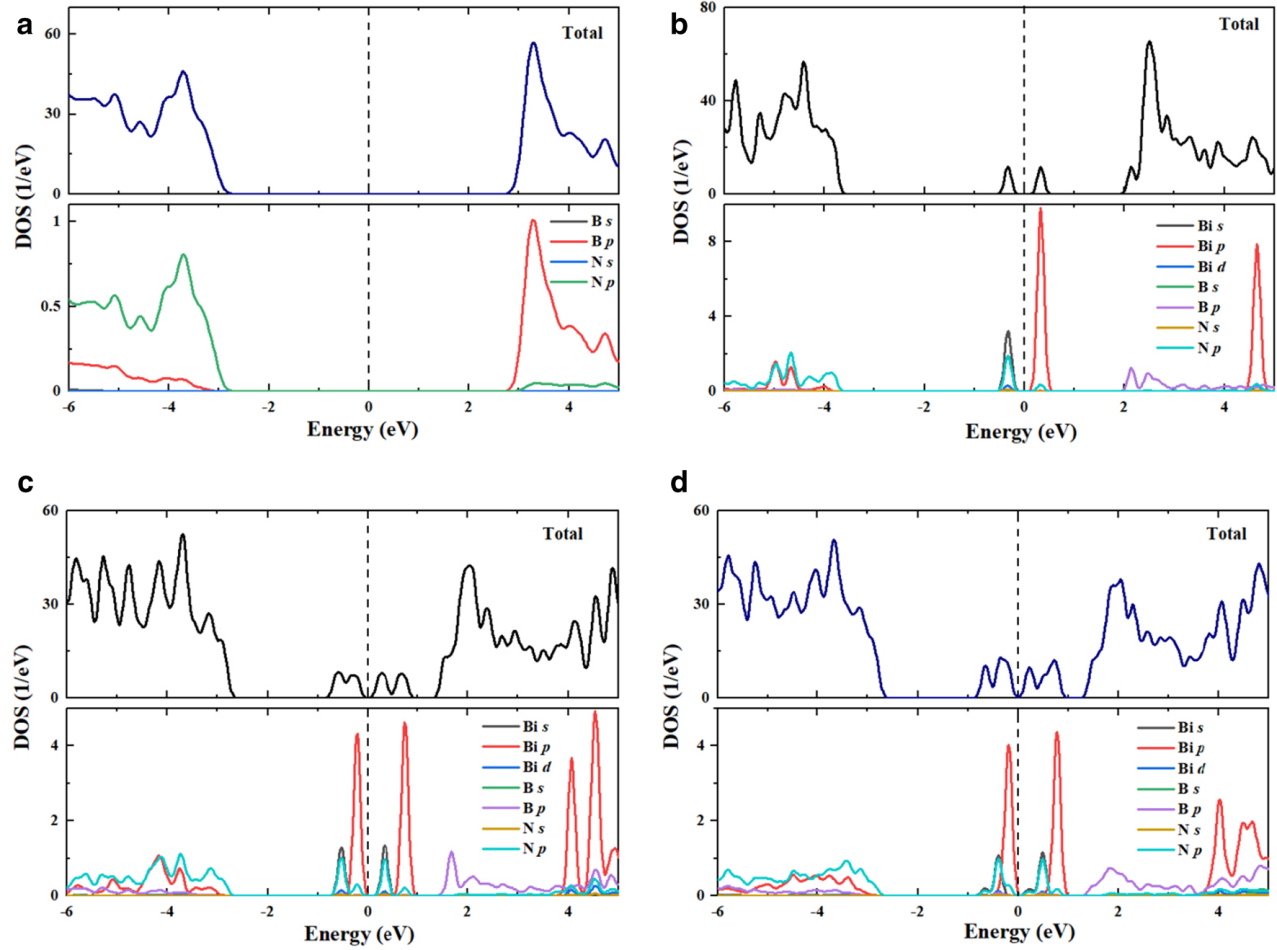
Calculated total and projected DOS of **a** pristine BN and Bi-doped BN monolayers with concentrations, **b** 2.04%, **c** 4.08%, and d) 6.1%

**Fig. 6:**
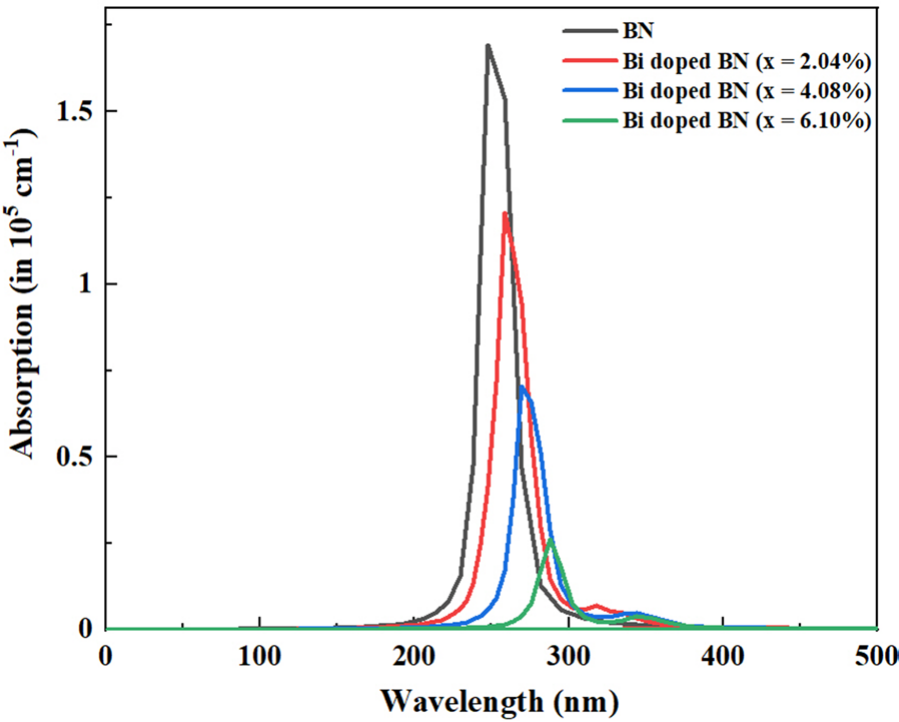
The simulated optical absorption spectra of pure and Bi-doped BN monolayers

To explore the impact of Bi dopants on the change of the electronic structures and the optical behavior, we computed the electronic band structures and density of states (DOS) by including SOC contribution of Bi-doped BN monolayer with different concentrations as well as pristine BN monolayer for comparison, as shown in Figs. 5 and 6. It can be seen that pristine BN has direct bandgap energy of magnitude 4.69 eV at K point, as shown in Fig. 4a, which is more consistent with measured experimental value (5.85 eV). In addition, our computed bandgap energy value is excellent agreement with previous theoretical works [72, 73]. This bandgap energy value indicates that BN monolayer is an insulator. According to the plots of the DOSs of the pristine from Fig. 5a, the valence band maxima is mainly featured by the N *2p* states whereas the minimum of the conduction band is mostly controlled by unoccupied B *2p* states. When introducing a Bi dopant with doping level of 2.04%, two new localized gap states are formed around Fermi level, as shown in Fig. 4b, in which the lower band is occupied whereas the upper band is unoccupied. Therefore, the valence band maximum has been shifted to lower in the valence band reducing the bandgap energy. Moreover, the main feature of the valence band maximum and conduction band minimum is similar to that of the pure BN monolayer. The appearance of impurity bands divides the bandgap energy into three energy sub-gap region having widths 3.39, 1.83 and 0.643 eV. The partial DOSs analysis (see Fig. 5b) reveals that the occupied gap states are mainly built up from Bi *6s* states mixed with N 2*p* states while the unoccupied gap states are mainly owing to Bi *6p* states with small contribution of N *2p* states. In the case of two Bi atoms doping into BN monolayer, the band structure presents a relatively larger shift downward of the conduction band. It is noticed that the number of dopant bands are increased causing further reduction in bandgap energy. The result is that Bi-doped BN monolayer exhibits typical characters of n-type semiconductor. It follows from Fig. 4c that four gap states have been introduced around Fermi level. The lowest two dopant levels are occupied and located about 0.57 and 0.21 eV below the Fermi level. The other two-gap states are unoccupied and situated at 0.40 and 0.80 eV above the Fermi level. However, the PDOS in Fig. 5c shows a large part of hybridization between Bi *6s* and N *2p* states for two small peaks and a great contribution of Bi *6p* states with a small contribution of N *2p* states for two high peaks. With increasing doping concentration of Bi at 6.1%, it is observed that more impurity states have been introduced around the Fermi level with reducing the bans gap, as shown in Fig. 4d. The impurity bands with lower energy gap states occur at 0.36 eV while the impurity bands with higher energy gap states locate at 0.61 eV above the Fermi level. From the plot of partial DOS (see Fig. 5d), it can be perceived for Bi doping in BN at concentration of 6.1% that the main feature of impurity bands below and above the Fermi level is similar to that of the Bi-doped BN with *x* = 4.08% with some overleaping between impurity bands below and above the Fermi level.

The absorption coefficients of pristine and Bi-doped BN monolayers are calculated and plotted in Fig. 6. It can be observed a redshift of the absorption edge with the increase of Bi doping concentration. In the Bi dopant concentration of 2.04%, the absorption edge shows a redshift of about 10 nm compared to that of pristine BN monolayer. This little redshift may appear from the slight bandgap energy decreasing and is in fair agreement with experimental measurement redshift of 20 nm for Bi doping into BN nanosheet. When the Bi incorporation concentration rising to 4.08% and 6.10%, the principal absorption edge has a more redshift about 22 nm and 40 nm compared with that of undoped BN monolayer. This has also resulted from the narrowing of bandgap energy, which leads to reproduce the experimental observation (see Fig. 3a). It can be observed that another very small absorption peak around 330 nm has appeared with the rise of Bi incorporation concentration. It further redshifts the absorption edge of the Bi-doped BN monolayer to a wavelength value of 345 nm (or energy of 3.60 eV), which signifies the enhancement of the catalytic ability.

### Catalytic Activity

Results of experiments to evaluate performance of catalytic activity of the synthesized material are represented by utilizing time-dependent UV–Vis spectra. It was observed that incorporation of reductant into an aqueous solution of dye was unable to degrade it as only ~ 7% of dye reduction was achieved. Addition of Bi-doped into BN nanosheets (nanocatalyst), percentage degradation is effectively enhanced. Pure BN nanosheets display 45% dye reduction in 35 min while BN doped with various concentrations (2.5, 5, 7.5, and 10 wt%) of Bi exhibit enhanced dye reduction with rapid progress.

**Fig. 7:**
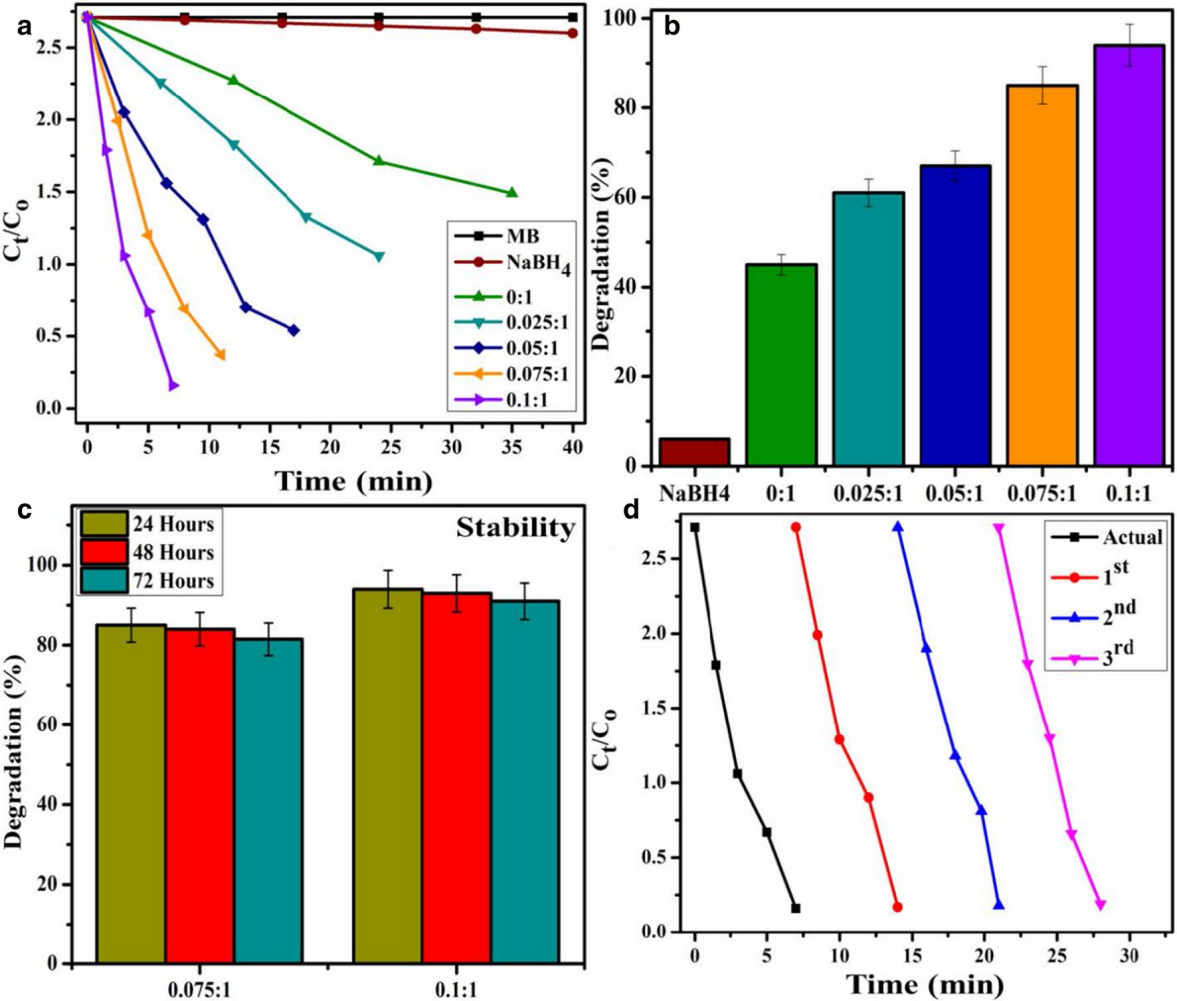
**a** Plots of *C*_*t*_/*C*_*o*_ versus time for all catalysts; **b** comparison of degradation percentage over various concentrations, **c** comparison of stability for 7.5 and 10 wt% Bi-doped catalyst; **d** plot of *C*_*t*_/*C*_*o*_ versus time for reusability of 10 wt% Bi-doped BN catalyst

In general, catalyst lowers the activation energy of a reaction which in turn causes to accelerate its stability and rate of reaction. MB is primarily a synthetic dye that is exploited into water during various industrial processes. MB can be reduced in the presence of reductant however the reduction process is relatively slow in the presence of only NaBH_4_. Host BN nanosheets exhibit large specific surface area causes to increase adsorption rate. Furthermore, a layer of reductant dispersed over nanocatalysts may also accelerate adsorption due to the redox reaction between catalyst and MB. Reduction reaction by a catalyst occurs by transferring an electron from donor species BH_4−_ (from NaBH_4_) to acceptor species MB facilitated by pure and Bi-doped BN nanosheets. This resulted to reduce activation energy which serves to stabilize and accelerate rate of the reaction [26, 74]. The mechanism of catalytic activity has been represented in Additional file 1: Fig. S7b. Dye degradation of various doped concentrations (2.5, 5, 7.5 and 10 wt%) was 61, 67, 85 and 94% in 24, 17, 11 and 7 min, respectively as illustrated in Fig. 7. The comparison of present experiment with literature is represented in Table 1.

**Table 1:** Comparison of dye degradation with respect to reported literature

Reported	Samples	Type	Efficiency
Dan Liu et al. [76], Xiaomeng et al. [77]	BN/TiO_2_ hybrid nanosheetsAg_3_PO_4_/h-BN composite	Degradation of Rhodamine Bdegradation of MB	Maximum degradation in 6 h Maximum degradation in 50 min
Man Du et al. [78]	Co@BN	Reduction towards 4-nitrophenol	Maximum degradation in 22 min
Jia Yan et al. [79]	WO_3_/h-BN nanocomposites	Degradation of Rhodamine B	Maximum degradation in 6 h
Present study	Bi-doped BN nanosheets	Degradation of MB	Maximum degradation in 7 min

Increase in the efficiency of catalytic activity is due to an increase in Bi concentration. As degradation percentage directly corresponds to the transfer of electrons from reducing agent towards MB which is facilitated by nanocatalyst. Bi-doped BN nanosheets cause to boost up the reaction rate by lowering its activation energy that in turn causes to facilitate transfer of electron more rapidly towards MB. Plot of *C*_*t*_/*C*_*o*_ as a function of time represents dye reduction of all samples as illustrated in Fig. 7a where *C*_*t*_ represents concentration of MB at any given time while *C*_*o*_ corresponds to initial concentration. Figure 9b exhibits degradation percentage of catalysts which was estimated through Eq. 3.

3 $$\mathrm{Degradation }(\mathrm{\%}) =\frac{Co-Ct}{Co}\times 100$$

Various factors that influence catalytic activity and affect the performance of catalysts are discussed below.

#### pH Value

In catalysis (catalytic activity), rate of reaction has a strong correlation with pH value. In general, an extremely low or high value of pH cannot contribute to dye degradation. Literature studies of catalytic activity using reducing agents demonstrate that rate of reaction at basic conditions is most favorable for maximum degradation. In the present study, the pH value at which the maximum degradation was attained was 8.4, which favorably correlates with literature cited. Further, materials such as BN nanosheets controls surface charge and dominate the possible electrostatic interaction between pollutant and material. Therefore, pH value of solution has direct linkage with removal process of pollutants by means of controlling the possible electrostatic interaction between the pollutant and adsorbent [74, 75].

#### Stability

The stability of catalyst was investigated in the present study by allowing performed experiment to stay for at least three days in order to examine whether the reduction of dye as performed in the presence of nanocatalyst is stable or not. In this regard degraded dye was kept in dark and after every 24 h, degradation was inspected with the help of absorption spectra acquired through UV–Vis spectrophotometer, as illustrated in Fig. 7c. Obtained results indicate that no loss of degradation occurred in stay condition for 72 h. Degradation was observed to be in its fairly original form which affirms the stability of catalyst.

#### Reusability

Reusability of catalyst refers to recycling ability of catalyst that permits its use more than once. Typically, catalysts with the most number of reusable cycles are considered the most efficient catalyst. In the current experiment, reusability was probed by recycling 10 wt% catalyst up to three cycles. The obtained results are presented in Fig. 7d, which indicates Bi-doped BN catalyst can be utilized as an effective reusable catalyst.

#### Load of Catalyst

Lastly, load of catalyst before the experiment and after three times of recycling was found. Load of catalyst before performing activity was 4 mg, after three times recycling it was measured as 3.7 mg, considering 5% sensing/detecting deviation. The results indicated that Bi-doped BN acts as the most stable, reusable, and the most efficient dye degrading catalyst. Furthermore, a load of catalyst after three days stability test was also performed that indicate almost same result (3.6 mg) as performed after recycling process.

### Bactericidal Activity

**Fig. 8:**
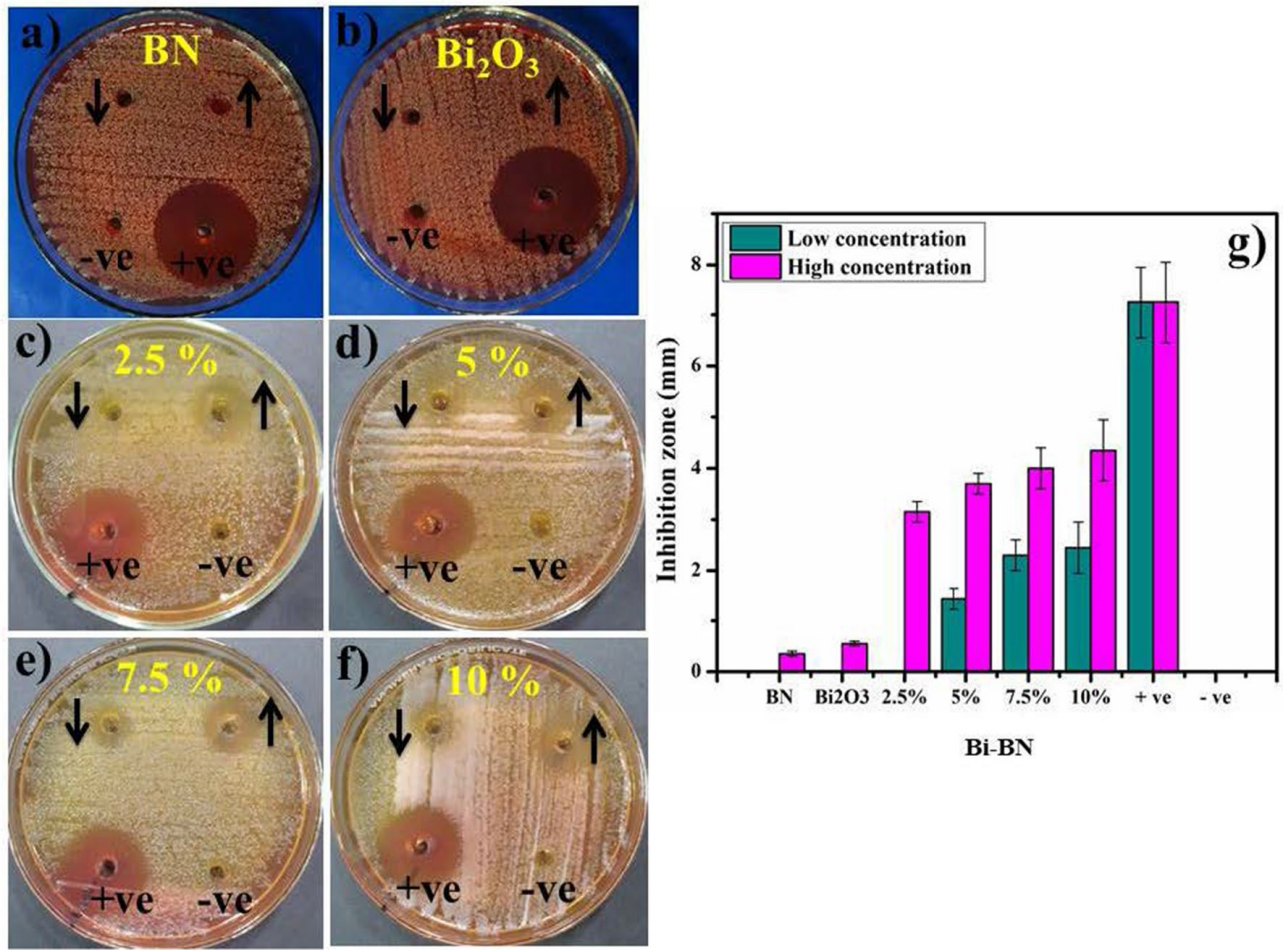
**a**–**g** In-vitro antimicrobial efficiency of BN (**a**) Bi_2_O_3_ (**b**) BN nanosheets doped with various concentrations (2.5, 5, 7.5 and 10 wt%) of Bi against *S. aureus* (**c**–**f**) graphical presentation (**g**)

**Fig. 9:**
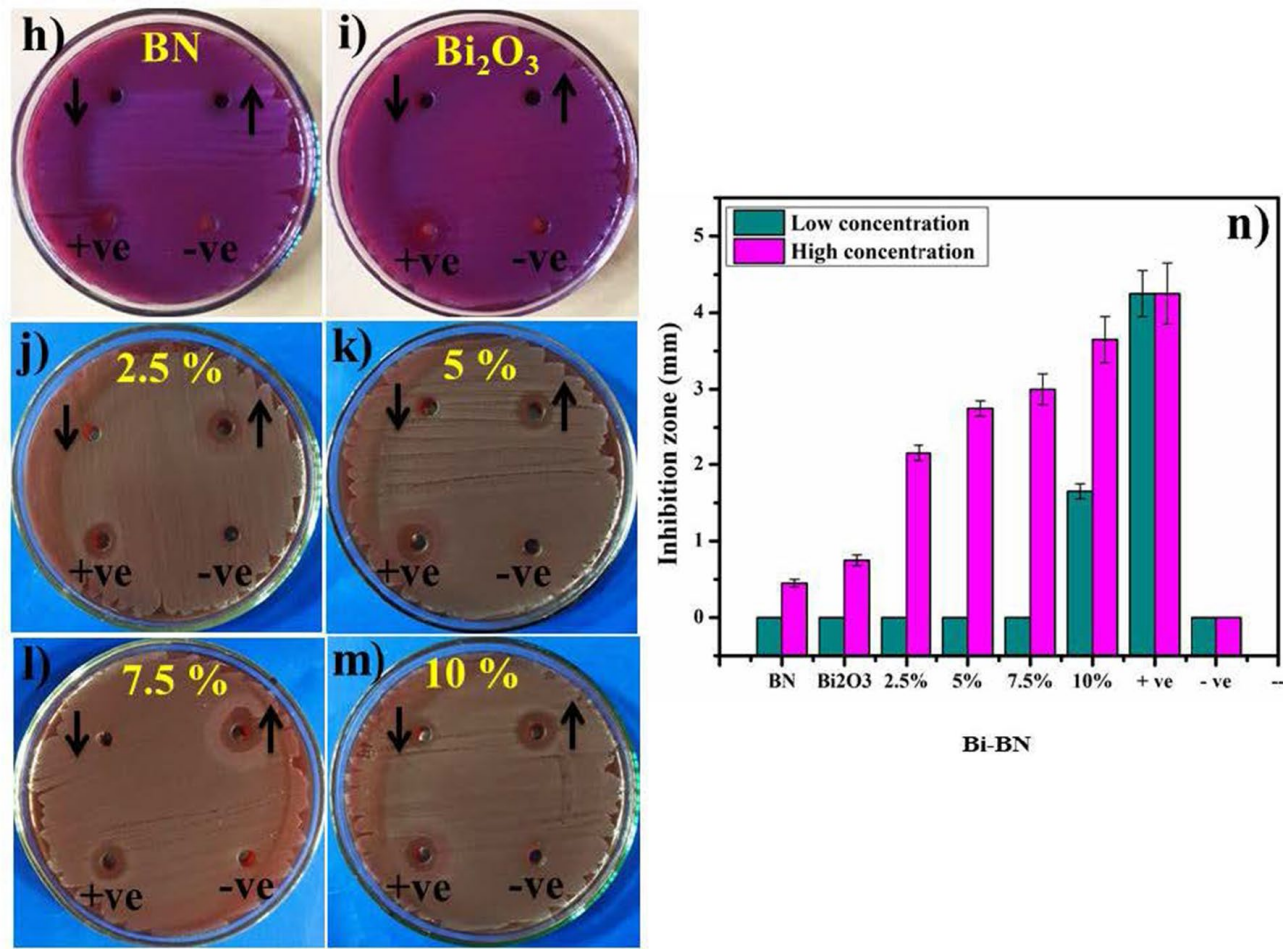
**h**–**n** In-vitro antimicrobial efficiency of BN (**h**) Bi_2_O_3_ (**i**) BN nanosheets doped with various concentrations (2.5, 5, 7.5 and 10 wt%) of Bi against *E. coli* (**j**–**m**) graphical demonstration (**n**)

*In-vitro* bactericidal activity of BN, Bi_2_O_3,_ and Bi-doped BN nanosheets for Gram + ve and Gram –ve bacteria are shown with graphical presentations in Figs. 8 and 9 (a–n). The findings indicate superior bactericidal action with synergism of Bi-doped BN nanosheets against *E. coli* compared with *S. aureus* as shown in Figs. 8 and 9 (a–j). BN and Bi_2_O_3_ at low concentrations showed null efficiency against G + ve and –ve bacteria. At high concentrations, BN depicted (0.35 mm) and (0.45 mm) inhibition and similarly, Bi_2_O_3_ showed (0.55 mm) and (0.75 mm) zone of inhibition against G + ve and –ve bacteria respectively Figs. 8 and 9 (a, b, h–i). Significant (*P* < *0.05*) inhibition zones were detailed as (0–2.45 mm) and (3.15–4.35 mm) for *S. aureus* and (0–1.65 mm) and (2.15–3.65 mm) for *E. coli* at low and high doses, respectively Figs. 8 and 9 (c–f, j–m). The efficiency percentage (% age) raised from (0–33.8%) and (43.4–60%) against *S. aureus* and (0–38.8%) and (50.5–85.8%) against *E. coli*, respectively. Ciprofloxacin used as positive control reduced (7.25 mm) and (4.25 mm) G+ve and –ve growth, respectively in comparison with DIW (0 mm). Generally, 2.5 wt% doped BN nanosheets showed zero efficiencies against Gram + ve and –ve bacteria at low dose while, other doped nanosheets depicted significant (*P* < *0.05*) antibacterial activity against Gram –ve compared to Gram +ve as shown in Figs. 8 and 9 (c–f, j–m).

The oxidative stress fashioned by nanosheets depends upon its size, shape, and concentration. Antibacterial activity with inhibition zones (mm) raised with greater wt% doping of Bi on BN due to more cationic availability. Antibacterial activity depending on size and concentration exhibited inverse relation to doped NS size [2, 22, 80]. ROS generation is considered a major hazardous factor for the destruction of micropathogens [81]. Small-sized NS produce reactive oxygen species (ROS) that stay more real within bacteria membrane within implants ensuing extrusion of cytoplasmic contents and damaging bacterial DNA, proteins, and enzymes thus, killing bacteria as illustrated in Additional file 1: Fig. S7 (a) [80, 82]. Upon irradiation, NPs activate e^−^ transfer from valence to conduction bands for reduction reactions by generating holes (h^+^) which, ultimately transfer to valence band for oxidation [83, 84]. The reduction process generates O_2_^−^. by reaction of e^−^ with O_2_ [85]. The holes (h^+^) via oxidation process generate OH through reaction with either e^−^ from water (H_2_O) or hydroxyl ions (OH^−^) [86]. The intense reactive oxygen radical species OH quickly reacts with micropathogens biomolecules i.e. proteins, carbohydrates, DNA, lipids and amino acids as shown in Additional file 1: Fig. S7 (a) [87]. Bismuth composites are famous for much effective antibacterial action coupled with low environmental toxicity [88]. Secondly, strong cationic interface of Bi^+3^ with negatively charged bacterial cell membrane parts grades in increased antibacterial action at high concentrations prompting bacteria collapse [2].

Enzyme catalyzing key steps of various biochemical reactions needed for bacterial survival represents attractive targets for antibiotic discovery. Molecular docking studies to predict inhibition tendency of nanoparticles against selected enzyme targets are of utmost importance for new antibiotic discovery. The mechanism of enzyme inhibition is depicted in Additional file 1: Fig. S7 (c) showing blockage of enzyme active site that hinder substrate access and disrupt catalytic activity of given enzyme target causing bacterial death.

Although extensive literature is reported over biological potential of nanomaterials particularly, bactericidal activity still clear mechanism of their action is not known. Nanomaterials show their antibacterial activity either through cell wall rapturing or may target key enzymes of various pathways that are essential for bacterial survival (see Additional file 1: Fig. S7) [80, 89]. Identifying their target is of worth importance and may contribute towards discovery of new antibiotics with a novel mode of action [90]. Here, enzyme targets of two well-known antibiotics i.e. Rifampicin (Nucleic acid synthesis) and Trimethoprim (Folate biosynthetic pathway) [91] have been selected to evaluate binding tendency, binding interaction pattern, and inhibitory mechanism of Bi-doped BN nanosheets behind their antibacterial activity.

**Fig. 10:**
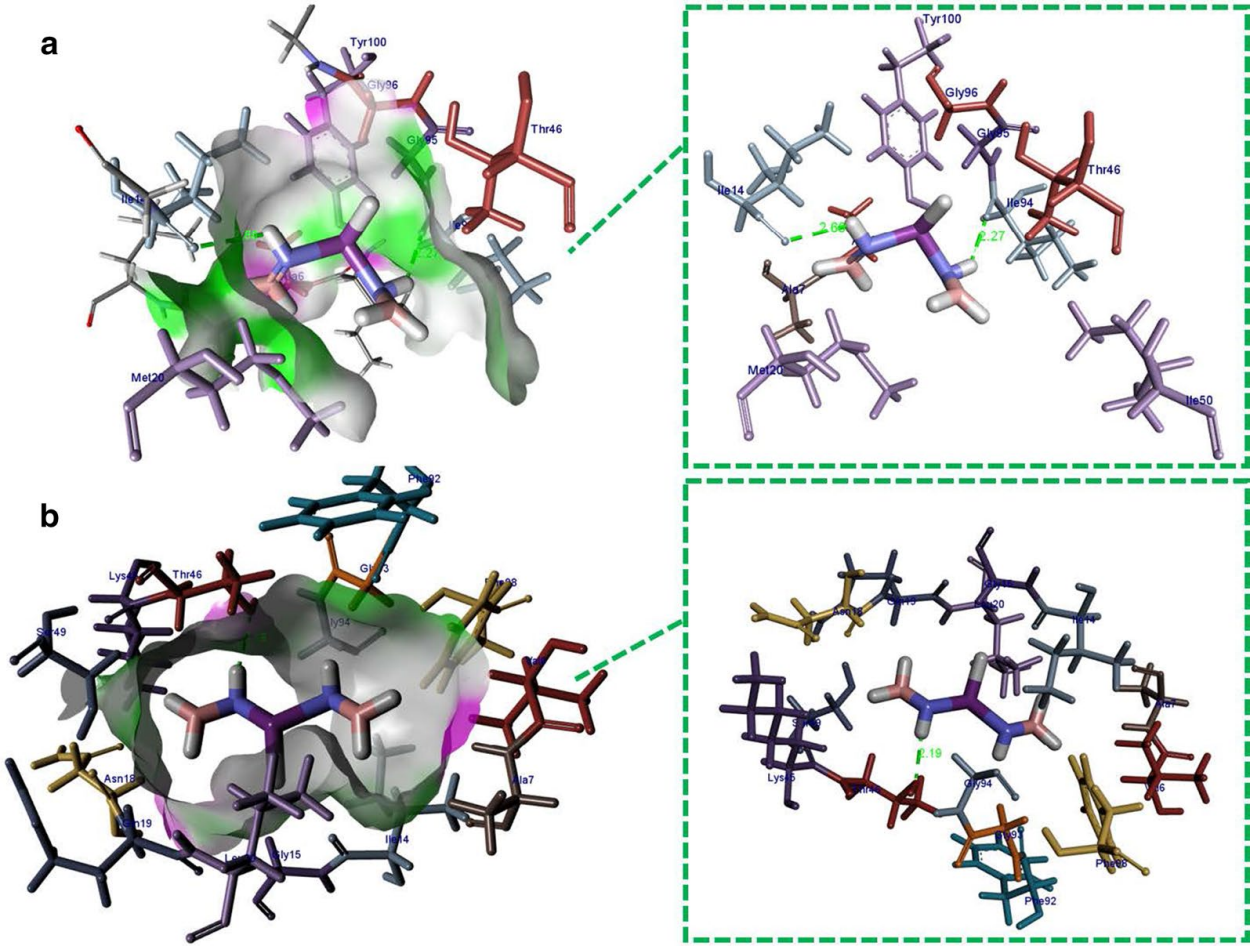
Binding interaction pattern of Bi-doped BN nanosheets with active site residues of dihydrofolate reductase from **a** *E. coli* and **b** *S. aureus*

In case of DHFR from *E.coli*, the best-docked conformation showed H-bonding interaction with Ile14 (2.68 Å) and Ile94 (2.27 Å) alongside metal-contact interaction with Tyr100 having binding score as − 11.971 kcal/mol (Fig. 10a). Similarly, H-bonding interaction with Thr46 (2.19 Å) and metal-contact with Leu20 was observed in case of DHFR from *S. aureus* having binding score − 8.526 kcal/mol as shown in Fig. 10b.

**Fig. 11:**
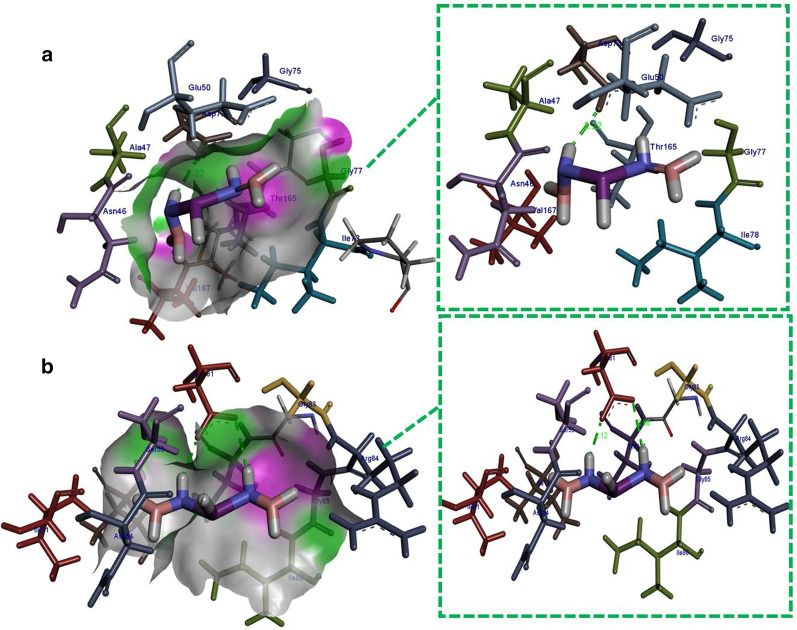
Binding interaction pattern of Bi-doped BN nanosheets with active site residues of DNA gyrase from (**a)** *E. coli* and **(b)** *S. aureus*

For DNA gyrase from *E.coli*, the best binding score observed was − 6.782 kcal/mol having H-bonding interaction with Asp73 (2.22 Å) as shown in Fig. 11a while in case of DNA gyrase from *S. aureus* H-bonding interaction were observed with Asp81 (2.12 Å and 2.68 Å) alongside metal contact interaction with Ile175 having binding score − 7.819 kcal/mol (Fig. 11b).

## Conclusion

BN nanosheets were successfully prepared through liquid-phase exfoliation of bulk BN while Bi was incorporated as dopant via hydrothermal approach. Various properties of synthesized material were studied using number of characterization approaches that are well harmonized with literature. XRD patterns indicated the presence of hexagonal phase of BN with peak shift to higher diffraction angle, which authenticates successful incorporation of dopant. FTIR spectra affirm the presence of in-plane B–N bending and out-of-plane B–N–B stretching vibrations, which corresponds to the presence of infrared active *E*_1u_ and *A*_2u_ modes of BN. The presence of luminescence band was affirmed through PL analysis whereas UV–Vis spectroscopy indicates the occurrence of absorption in near UV region. Morphological examinations were studied via FESEM and HR-TEM micrographs indicated sheet-like morphology with decoration of Bi over nanosheets, which signifies an effective doping procedure. Interlayer spacing estimated through HR-TEM images with the aid of Gatan digital micrograph software that corresponds well with XRD; while EDS spectra showed strong signals originating from both pure as well as dopant material. The optimization results from the first principle calculation reveal that Bi can be substituted and stable into BN nanosheets with different concentrations. Impurity bands due to Bi atoms introduce a sub-bandgap energy absorption in the electronic bandgap energy region which might increase the catalytic activity. Investigation of dye degradation via CA experiments resulted in an efficient and rapid process. Further pure and doped BN nanosheets serve as stable, reusable, and outstanding nanocatalyst for wastewater treatment. In addition, antimicrobial efficiency of doped BN nanosheets against *S. aureus* and *E. coli* isolated directly from caprine mastitic milk resulted in significant quantitative values. In silico predictions against selected enzyme targets i.e. DHFR and DNA gyrase from *E. coli* and *S. aureus* were in good agreement with in-vitro bactericidal activity thereby, opening a new horizon for the use of doped nanomaterials as potential agents for antimicrobial and CA procedures. Theoretical calculations are in good agreement with experimental values. Theoretical study indicates that substitutional doping of Bi with different concentrations is stable. Moreover, Bi doping led to various modifications in the electronic structures of BN nanosheets by inducing new localized gap states around the Fermi level. Finally, upon these results, it can be concluded that Bi-doped BN nanosheets is a suitable material to utilize in industrial wastewater applications, and antimicrobial treatment.

## Supplementary Information

**Additional file 1**. Supplementary Materials.

## Data Availability

All data are fully available without restriction.

## Abbreviations

Bi: Bismuth
BN: Boron nitride
EDS: Energy-dispersive X-ray spectroscopy
FESEM: Field emission scanning electron microscopy
FTIR: Fourier transform infrared spectroscopy
G+ve: Gram-positive
G–ve: Gram-negative
HR-TEM: High-resolution transmission electron microscope
JCPDS: Joint Committee on Powder Diffraction Standards
MA: MacConkey agar
MB: Methylene blue
nm: Nanometer
PL: Photoluminescence
UV–Vis: Ultra-violet visible spectroscopy
XRD: X-ray diffraction
