# Supplementary material for: Impact of Bi Doping into Boron Nitride Nanosheets on Electronic and Optical Properties Using Theoretical Calculations and Experiments
Source: Nanoscale Res Lett. 2021 May 12;16:82. doi: 10.1186/s11671-021-03542-x (PMC8116421; doi:10.1186/s11671-021-03542-x)
Supplement: Supplementary file 1 — Additional file 1. Supplementary Materials. [file 11671_2021_3542_MOESM1_ESM.docx]

**Supplementary Materials**

**Impact of Bi doping into boron nitride nanosheets on electronic and optical properties using theoretical calculations and experiments**

M. Ikram^a*^, M. Wakeel^b^, J. Hassan^b^, A. Haider^c^, S. Naz^d^, A. Ul-Hamid^e^, J. Haider^d^, S. Ali^b^, S. Goumri-Said^f^, M. B. Kanoun^g*^

^a^Solar Cell Applications Research Lab, Department of Physics, Government College University Lahore, 54000, Punjab, Paksitan

^b^Department of Physics, Riphah Institute of Computing and Applied Sciences (RICAS), Riphah International University, 14 Ali Road, Lahore, Pakistan

^c^Department of Clinical Medicine and Surgery, University of Veterinary and Animal Sciences

Lahore, 54000, Punjab, Pakistan

^d^Tianjin Institute of Industrial Biotechnology, Chinese Academy of Sciences, Tianjin 300308, China

^e^Core Research Facilities, Research Institute, King Fahd University of Petroleum & Minerals, Dhahran 31261, Saudi Arabia.

*^f^ College of Science, Physics Department, Alfaisal University, P.O. Box 50927, Riyadh 11533, Saudi Arabia*

*^g^ Department of Physics, College of Science, King Faisal University, P.O. Box 400, Al-Ahsa, 31982, Saudi Arabia*

^*^Corresponding Author Email: [dr.muhammadikram@gcu.edu.pk](mailto:dr.muhammadikram@gcu.edu.pk), mkanoun@kfu.edu.sa

**1. Isolation and identification of *S. aureus* and *E. coli***

Clinically positive samples of caprine mastitic milk were gathered from various farms. Initially, samples were cultured at sheep blood agar (5%) and kept at 37 ˚C for twenty-four hours. The cultured bacterial growth was further streaked at mannitol salt agar (MSA) and MacConkey agar (MA) in triplets for isolation of purified *S. aureus* and *E. coli*, respectively. Morphological characterization of characteristic colonies thus obtained was carried through Gram’s staining and biochemically using catalase and coagulase tests [1].


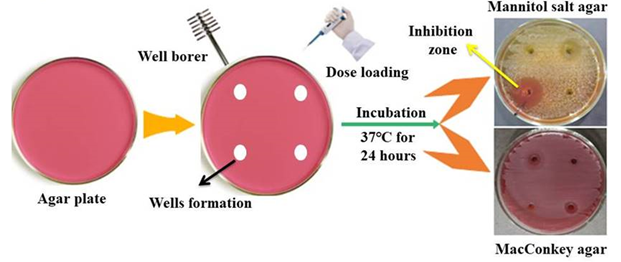


Fig. S1: Agar well diffusion method

**2. Statistical analysis**

The bactericidal response of Bi-doped BNNS as inhibition zones (mm) measurements was considered significant with a one-way analysis of variance (ANOVA) using SPSS 20 version [2].

**3. Molecular docking studies**

Advent of antibiotic resistance in last couple of decades signifies the need for more compatible and effective antibiotics agents. Dihydrofolate reductase (DHFR), a key enzyme of folate biosynthetic pathway represents an attractive target for antibiotics discovery since discovery of trimethoprim (TMP) [3, 4]. Similarly, inhibitors of DNA gyrase enzyme from nucleic acid biosynthetic pathway may serve as scaffolds for new antibiotics discovery [5]. Here, Molecular docking studies of Bi-doped BN nanosheets has been performed against DHFR and DNA gyrase from both *E. coli* and *S. aureus* to predict their binding tendency, mechanism of inhibition and mode of action behind bactericidal activity.

Selected protein’s structural information and 3D-coordinates were retrieved from protein data bank with accession codes for DHFR as 2ANQ; Res: 2.13 Å [6], 3M08; Res: 2.01 Å [7] from *E. coli* and *S. aureus*, respectively. Similarly, PDB ID for DNA gyrase crystal structure used were 6KZX; Res: 2.1 Å from *E. coli* and 5CTU; Res: 1.45 Å [8]from *S. aureus* given in Fig. S2.


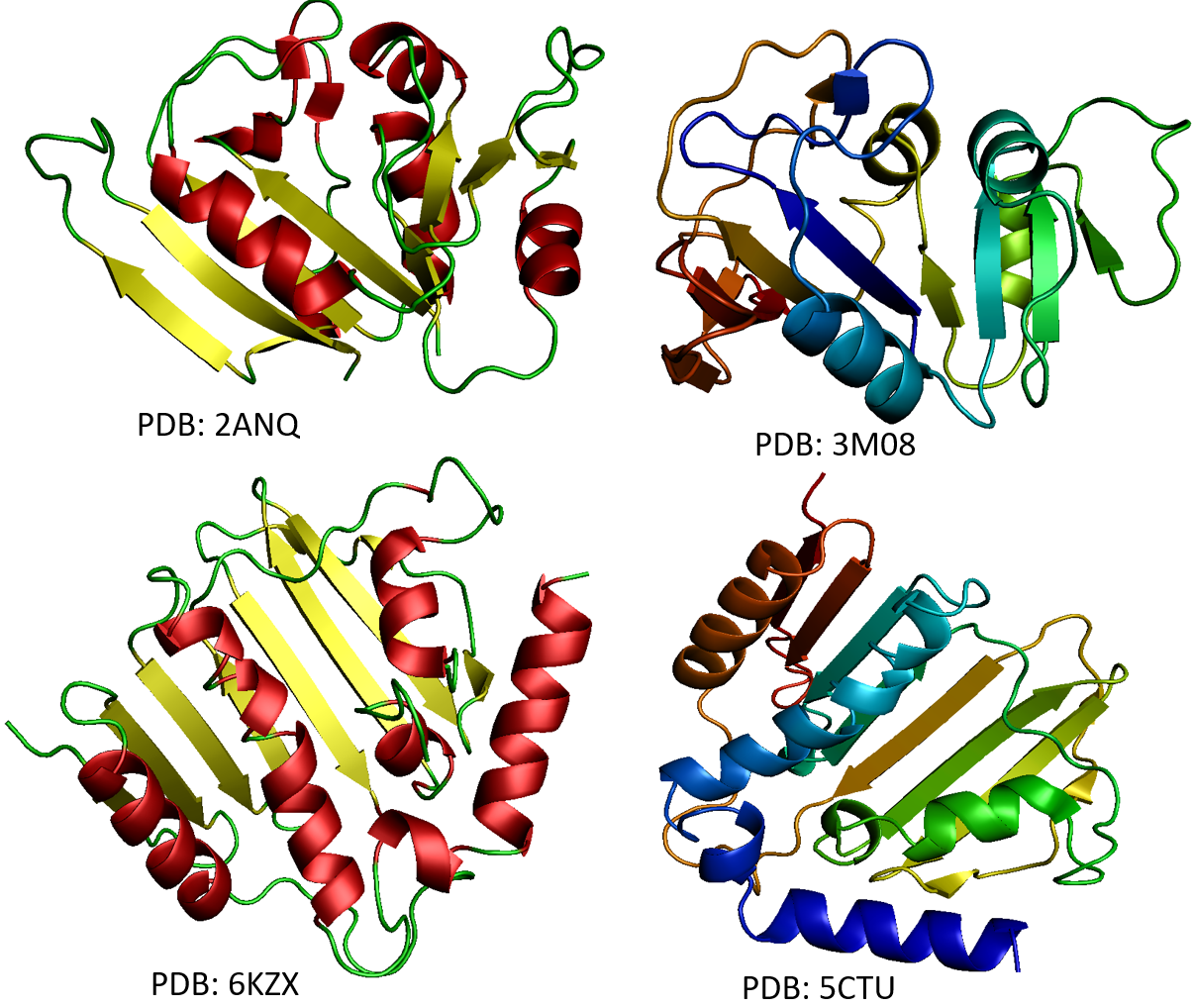


**Figure S2:** 3D-structure of Dihydrofolate reductase (DHFR) and DNA gyrase from *E. coli* and *S. aureus*

Molecular docking studies were performed using molecular operating environment (MOE) software [9]. Builder tool of MOE was utilized for ligand structure preparation while target protein’s structures were prepared using a multistep method. Various steps involved in protein structure preparation were removal of water molecules and co-crystallized ligands followed by energy minimization using MMFF94x force field and gradient: 0.00001. Later, binding pocket was defined around ligand (within 10 Å distance) of protein and best docked conformations with lowest energy were generated in each case. Binding interaction analysis and 3D visualization of best docked complexes were done using discovery studio visualizer [10] and Pymol software.

**4. Morphological analysis**


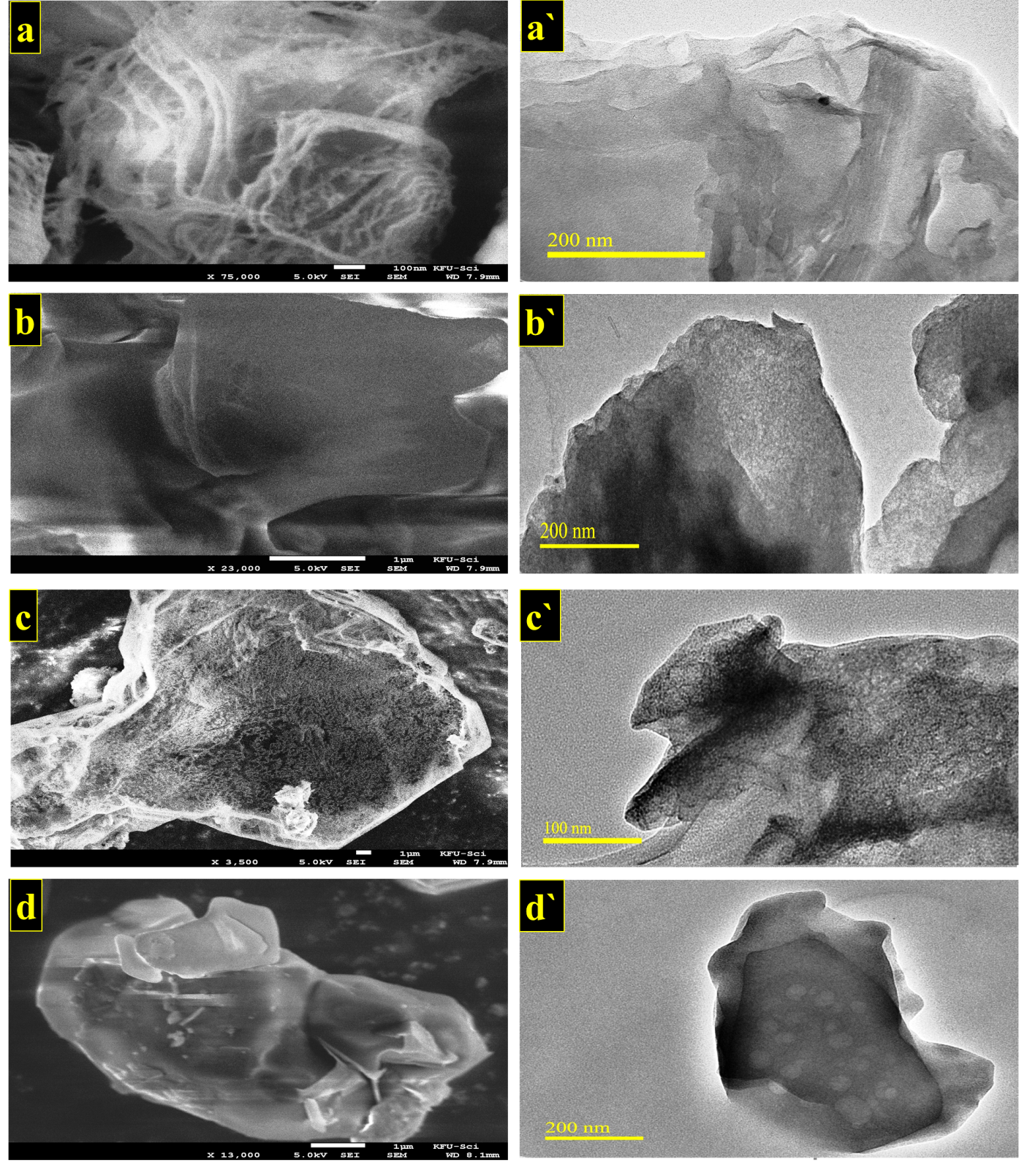


Fig. S3: (a-d) FESEM micrographs of BN nanosheets doped with various concentrations (0, 2.5, 7.5 and 10 wt.%) of Bi; (a’-d’) HR-TEM micrographs

Morphology of host and Bi-doped BN nanosheets was examined by means of FESEM as demonstrated in Fig. S3(a-d). Obtained images show that the samples possessed plate or sheet-like morphology with non-uniform compact features. Some agglomeration was also observed in all samples. Morphology of prepared product was also analyzed through HR-TEM analysis as displayed in Fig. S3(a’-d’). Nanosheets with slight porous features and faint transparency can be seen in obtained micrographs. Additionally, dark spots were observed decorating the synthesized material in HR-TEM images, which provide evidence for the presence of doping element on nanosheets. Dimensions and configuration of nanosheets indicate transformation of bulk BN to nanosheets as a result of exfoliation [11, 12].

**5. Interlayer spacing analysis**


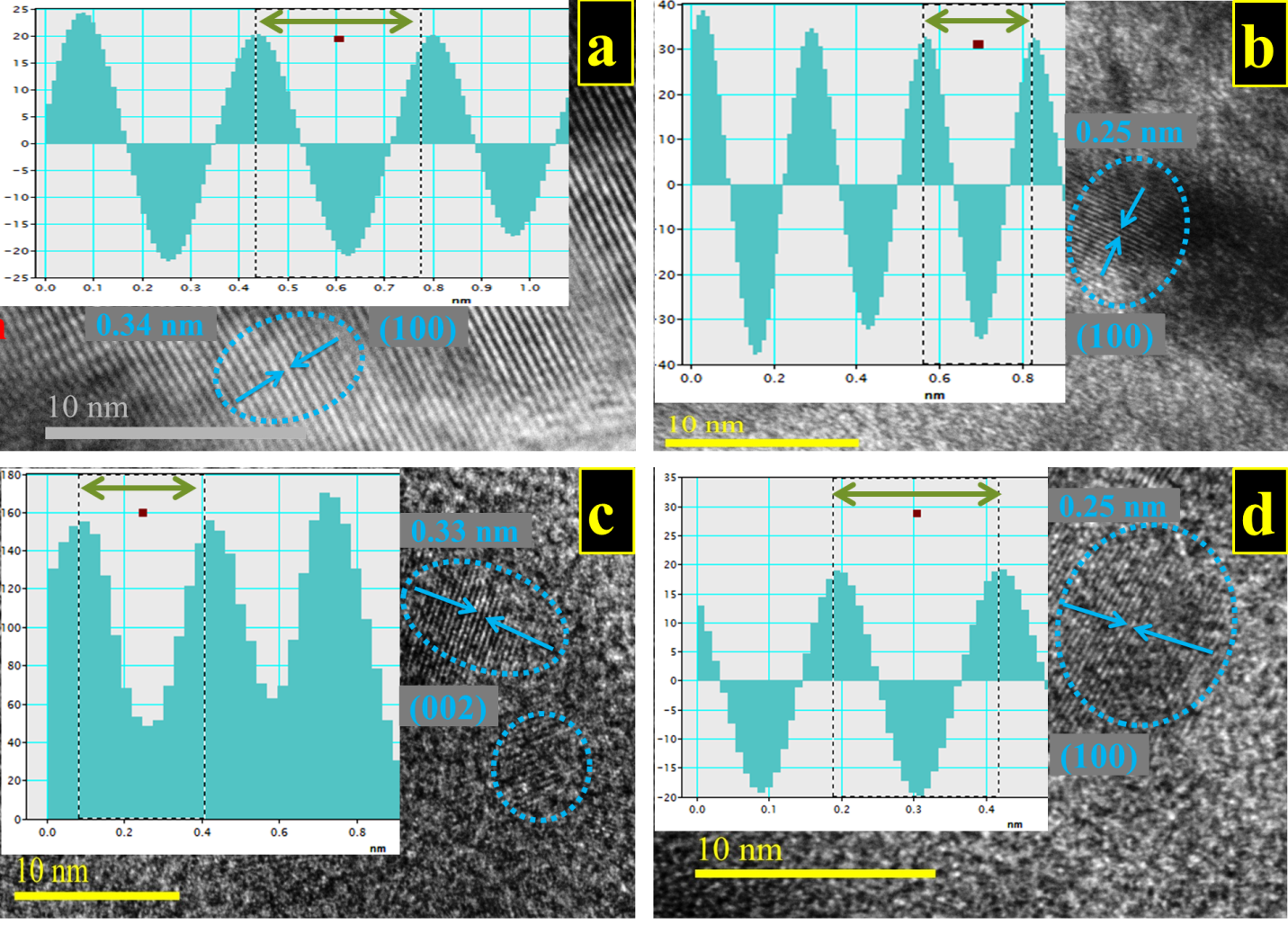


Fig. S4: (a) Interlayer spacing examination of host BN nanosheets; (b-d) BN nanosheets doped with various concentrations (2.5, 5, 7.5 and 10 wt%) of Bi.

Interlayer spacing was calculated from HR-TEM micrographs employing Gatan Digital Micrograph software which allows differentiation between lattice fringes; see Fig. S4(a-d). In case of control sample, d-value was found to be 0.34 nm which corresponds to (002) plane. Similarly, d-value calculated from doped NS was found to be 0.25 and 0.33 nm that correlate with (100) and (002) planes, respectively. These values match well with standard data reported in the literature [13].

**6. EDX analysis**


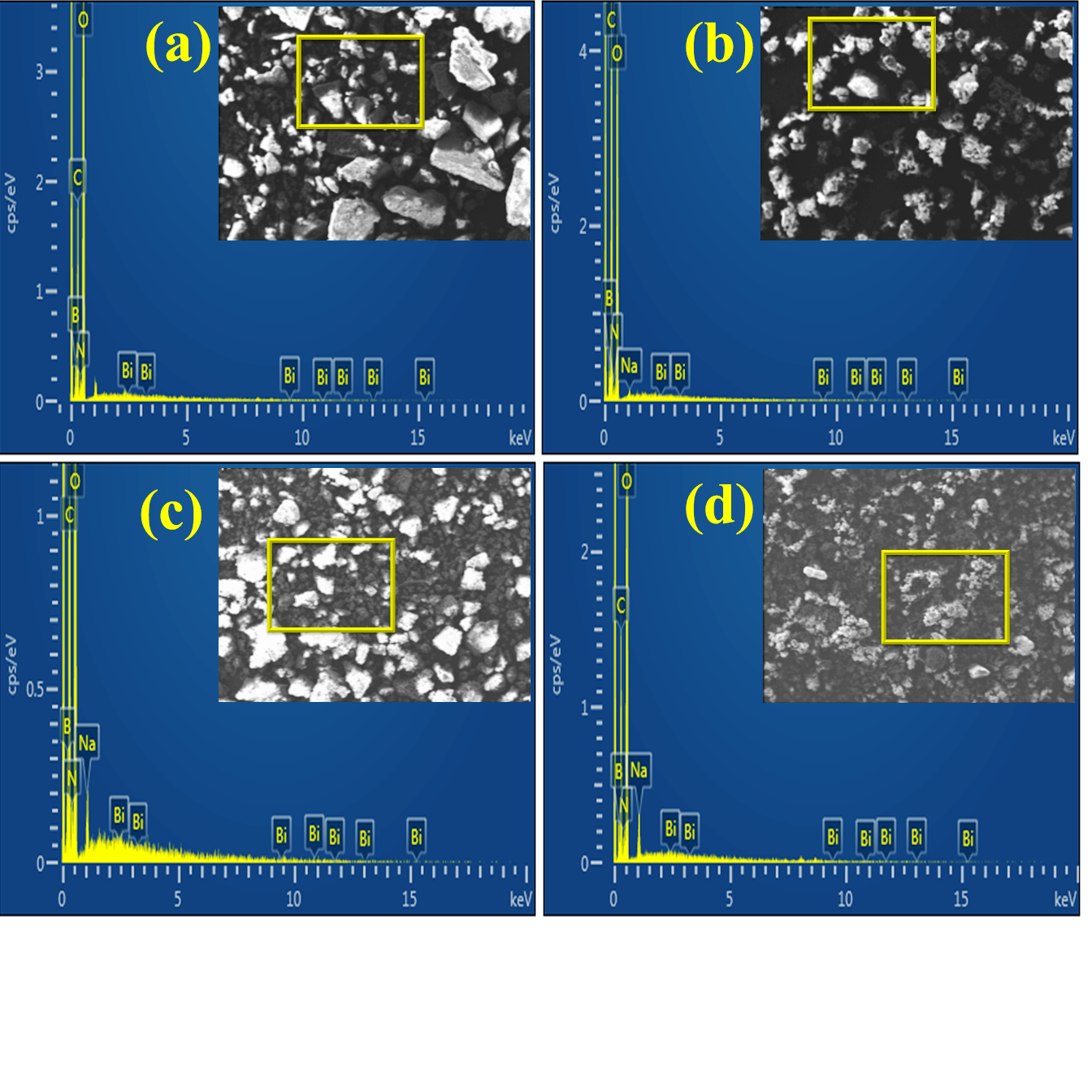


Fig. S5: (a-d) EDS examination of various concentrations (2.5, 5, 7.5 and 10 wt%) of Bi-doped BN nanosheets

Elemental composition and evidence of doping in BNNS were obtained using EDS analysis. Fig. S5 (a-d) represent EDS spectra acquired from BNNS s doped with various concentrations (2.5, 5, 7.5 and 10 wt%) of Bi. Strong peaks originating from host material (B & N) as well as dopant material (Bi) are detected in all samples, which indicate successful materials synthesis and effective incorporation of dopant. Additionally, certain peaks (C, O & Na) were also detected which may originate due to contamination. Sodium peak may originate due to contaminated sample holder while characterizing the material. Carbon peak stems from the carbon tab upon which the samples are placed during examination with SEM. There is no carbon in the sample [14].


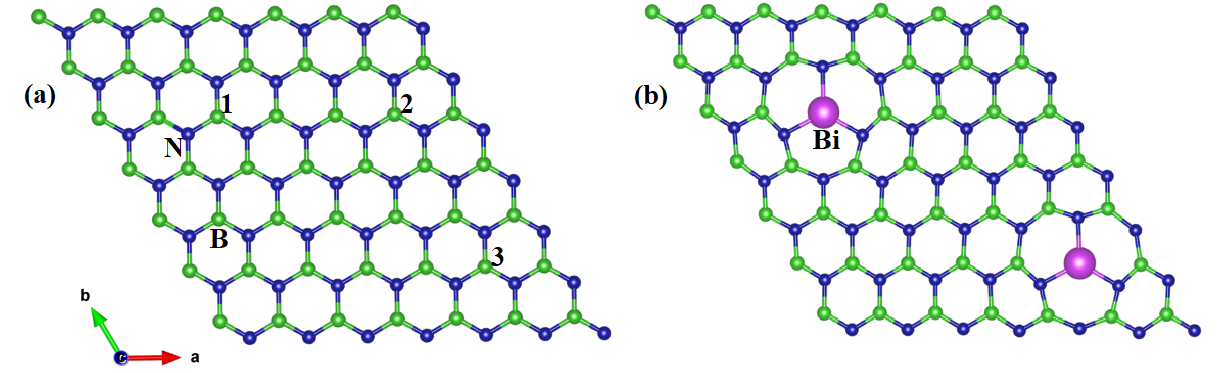


Fig. S6. (a-b) Schematic diagram of the possible doping sites of Bi atoms on monolayer BN, and green, Blue, and violet balls represent B atoms, N atoms, and Bi atoms, respectively


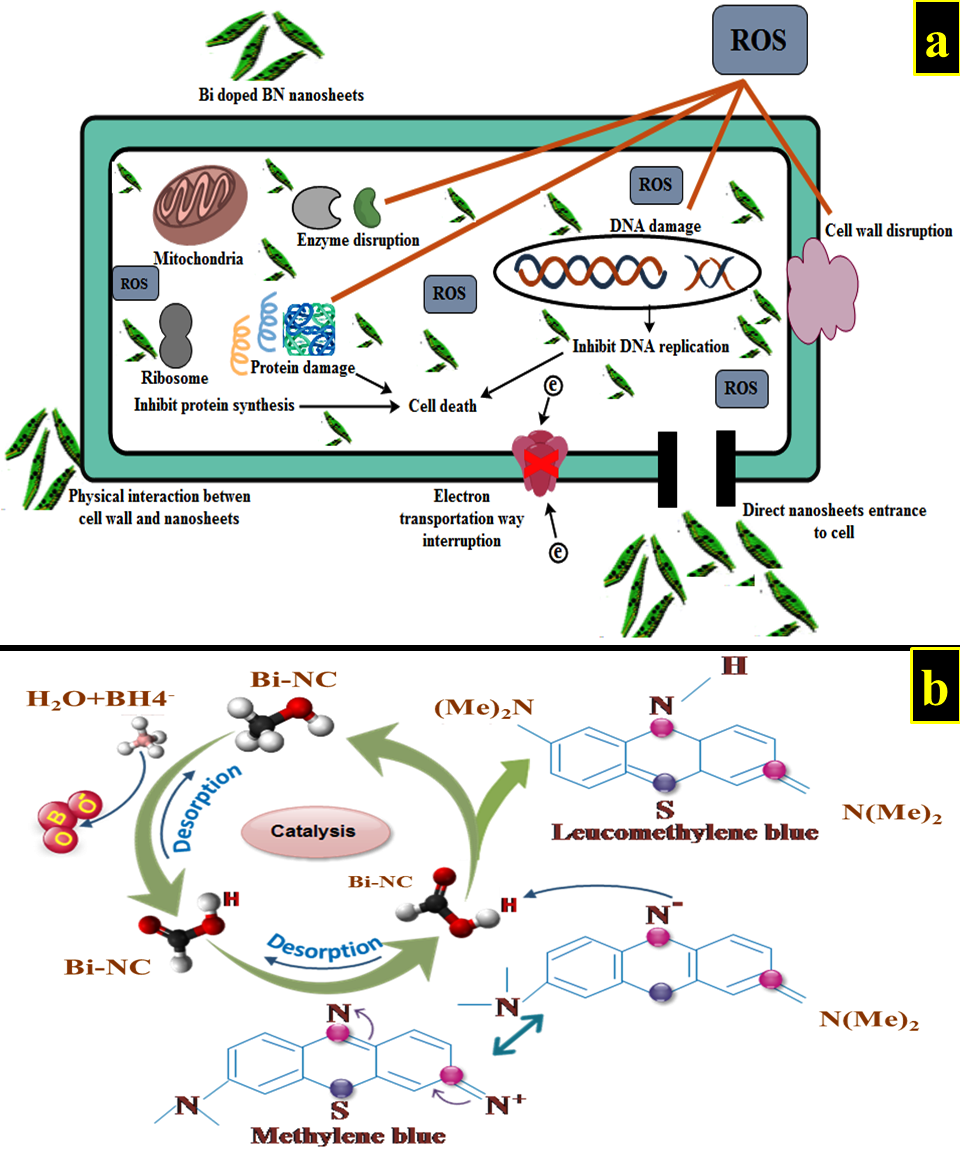

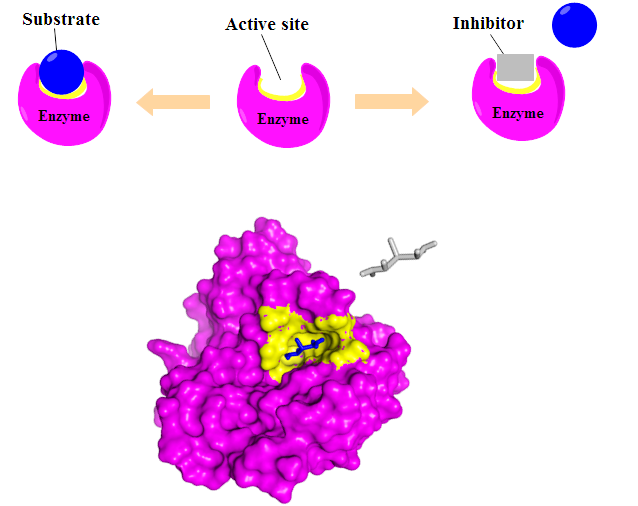


**C**

Fig. S7: Illustration of mechanism for (a) antimicrobial; (b) catalytic activity; (c) molecular docking studies

**References:**

1. Haider, A., et al., *Enhanced bactericidal action and dye degradation of spicy roots’ extract-incorporated fine-tuned metal oxide nanoparticles.* Applied Nanoscience, 2020. **10**(4): p. 1095-1104.

2. Haider, A., et al., *Green Synthesized Phytochemically (Zingiber officinale and Allium sativum) Reduced Nickel Oxide Nanoparticles Confirmed Bactericidal and Catalytic Potential.* Nanoscale Research Letters, 2020. **15**(1): p. 50.

3. Dale, G.E., et al., *A single amino acid substitution in Staphylococcus aureus dihydrofolate reductase determines trimethoprim resistance 1 1 Edited by T.Richmond.* Journal of Molecular Biology, 1997. **266**(1): p. 23-30.

4. Hitchings, G.H., *Mechanism of Action of Trimethoprim-Sulfamethoxazole—I.* The Journal of Infectious Diseases, 1973. **128**(Supplement_3): p. S433-S436.

5. *<ddd.pdf>.*

6. Summerfield, R.L., et al., *A 2.13 Å Structure of E. coli Dihydrofolate Reductase Bound to a Novel Competitive Inhibitor Reveals a New Binding Surface Involving the M20 Loop Region.* Journal of Medicinal Chemistry, 2006. **49**(24): p. 6977-6986.

7. Bourne, C.R., et al., *Inhibition of Antibiotic-Resistant &lt;em&gt;Staphylococcus aureus&lt;/em&gt; by the Broad-Spectrum Dihydrofolate Reductase Inhibitor RAB1.* Antimicrobial Agents and Chemotherapy, 2010. **54**(9): p. 3825.

8. Ushiyama, F., et al., *Lead Identification of 8-(Methylamino)-2-oxo-1,2-dihydroquinoline Derivatives as DNA Gyrase Inhibitors: Hit-to-Lead Generation Involving Thermodynamic Evaluation.* ACS Omega, 2020. **5**(17): p. 10145-10159.

9. Labute, P.J.I., Montreal, *Molecular Operating Environment. Chemical Computing Group.* 2008.

10. Systèmes, D.J.D.S.B.S.D., CA, USA, *Biovia, discovery studio modeling environment.* 2016.

11. Raza, A., et al., *A comparative study of dirac 2D materials, TMDCs and 2D insulators with regard to their structures and photocatalytic/sonophotocatalytic behavior.* Applied Nanoscience, 2020. **10**(10): p. 3875-3899.

12. Zhao, G., et al., *Large-quantity and continuous preparation of two-dimensional nanosheets.* Nanoscale, 2016. **8**(10): p. 5407-5411.

13. Li, C., et al., *Thickness-dependent bending modulus of hexagonal boron nitride nanosheets.* Nanotechnology, 2009. **20**(38): p. 385707.

14. Ikram, M., et al., *2D chemically exfoliated hexagonal boron nitride (hBN) nanosheets doped with Ni: synthesis, properties and catalytic application for the treatment of industrial wastewater.* Applied Nanoscience, 2020. **10**(9): p. 3525-3528.
